# The Resting Potential and K^+^ Currents in Primary Human Articular Chondrocytes

**DOI:** 10.3389/fphys.2018.00974

**Published:** 2018-09-04

**Authors:** Mary M. Maleckar, Robert B. Clark, Bartholomew Votta, Wayne R. Giles

**Affiliations:** ^1^Simula Research Laboratory, Center for Biomedical Computing and Center for Cardiological Innovation, Oslo, Norway; ^2^Allen Institute for Cell Science, Seattle, WA, United States; ^3^Faculty of Kinesiology, University of Calgary, Calgary, AB, Canada; ^4^Glaxo Smith Kline, Collegeville, PA, United States; ^5^Faculties of Kinesiology and Medicine, University of Calgary, Calgary, AB, Canada

**Keywords:** human chondrocyte, patch clamp recordings, K^+^ currents, TRP channels, mathematical model, resting membrane potential, depolarization-secretion coupling

## Abstract

Human transplant programs provide significant opportunities for detailed *in vitro* assessments of physiological properties of selected tissues and cell types. We present a semi-quantitative study of the fundamental electrophysiological/biophysical characteristics of human chondrocytes, focused on K^+^ transport mechanisms, and their ability to regulate to the resting membrane potential, E_m_. Patch clamp studies on these enzymatically isolated human chondrocytes reveal consistent expression of at least three functionally distinct K^+^ currents, as well as transient receptor potential (TRP) currents. The small size of these cells and their exceptionally low current densities present significant technical challenges for electrophysiological recordings. These limitations have been addressed by parallel development of a mathematical model of these K^+^ and TRP channel ion transfer mechanisms in an attempt to reveal their contributions to E_m._ In combination, these experimental results and simulations yield new insights into: (i) the ionic basis for E_m_ and its expected range of values; (ii) modulation of E_m_ by the unique articular joint extracellular milieu; (iii) some aspects of TRP channel mediated depolarization-secretion coupling; (iv) some of the essential biophysical principles that regulate K^+^ channel function in “chondrons.” The chondron denotes the chondrocyte and its immediate extracellular compartment. The presence of discrete localized surface charges and associated zeta potentials at the chondrocyte surface are regulated by cell metabolism and can modulate interactions of chondrocytes with the extracellular matrix. Semi-quantitative analysis of these factors in chondrocyte/chondron function may yield insights into progressive osteoarthritis.

## Introduction

Articular cartilage is a major component of the flexible connective tissue that covers the opposed ends of articular joints. It is essential for the stability and low friction movement of the associated long bones (Huber et al., 2000). This tissue is populated predominately by only one type of cell—the *chondrocyte*, and it lacks any significant vascular, or lymphatic elements. Each chondrocyte, together with its immediate pericellular coat or glycocalyx, forms a functional unit that has been named a “chondron” (Poole, 1997; Guilak et al., 2006; Nguyen et al., 2010; McLane et al., 2013). Under physiological circumstances, cyclical mechanical forces within the joint capsule create a dynamic environment that modulates cellular metabolism and maintains overall health (Wu and Chen, 2000; Guilak et al., 2006; Chen et al., 2013).

Although chondrocytes occupy only ~1–10% of the total volume of mammalian articular cartilage (Hall et al., 1996; Archer and Francis-West, 2003), they play essential roles in the homeostasis of the extracellular matrix (ECM). In part, this is because these cells synthesize and secrete most of the essential lubricants within the joint, including hyaluronan and lubricin (Ogawa et al., 2014). The ECM is composed of: (i) collagen fibers that give the tissue the ability to resist tension, (ii) negatively charged gel-like proteoglycans that are trapped within the collagen mesh and allow the tissue to bear compression, and (iii) synovial fluid which acts as a lubricant, thus ensuring low friction movement of the bones. The primary role of the chondrocyte is to maintain viable cartilage by regulating macromolecular synthesis and breakdown of its essential constituents and to produce lubricants (Huber et al., 2000; Guilak et al., 2006; Ogawa et al., 2014).

In a variety of progressive chronic diseases, or as a consequence of injury, there is chondrocyte damage and related dysfunction (Bush et al., 2003; Martin and Buckwalter, 2003; Bush and Hall, 2005; Mobasheri et al., 2015). In these situations, the dynamic balance between matrix synthesis and degradation is altered and the low friction environment within the joint may also be reduced (Urban et al., 1993). Frequently, there also is an inflammatory response within the articular joint (Pelletier et al., 2001). These factors can increase the early development of osteoarthritis, and attendant thinning of the cartilage layer, thus resulting in painful, bone-against-bone friction (Bush et al., 2003; Mobasheri et al., 2015). The progression of osteoarthritis and the reduced ability of chondrocyte ion transport systems to respond to perturbations in the extracellular environment (Pelletier et al., 2001) have also been associated with deficiencies in volume regulation (Urban et al., 1993; Lewis et al., 2011). It is known that damage to cartilage is more prominent in the setting of co-incident changes in osmolarity in the chondron. In part, this may be because these volume changes are linked to an abnormal resting membrane potential in chondrocytes (Lewis et al., 2011) caused by altered ion transport, e.g., changes in K^+^ and/or Cl^−^ channel activity. However, uncertainty and some disagreements remain concerning the fundamental ionic mechanisms for this progressive loss of function of the affected chondrocytes in chronic disease settings.

Detailed experimental investigations that address possible functional relationships between chondrocyte electrophysiology and pathophysiology are technically challenging. This is mainly due to the very small size of a mature chondrocyte and the associated limitations of *in vitro* electrophysiological/biophysical studies. In fact, it is not certain that conventional patch pipette methods (Lewis et al., 2011) can accurately determine the resting potential of isolated single chondrocytes (Ince et al., 1986; Mason et al., 2005; Wilson et al., 2011). Partly for this reason, and also to allow us to integrate our patch clamp results with other experimental data we have developed a mathematical model based on the fundamental components responsible for K^+^ transport in the human chondrocyte. This model is based mainly on experimental data obtained from human chondrocyte preparations.

The goals of this paper are: (i) to identify the main K^+^ currents that contribute to the resting membrane potential (ii) to develop the first mathematical model of essential electrophysiological principles exhibited by human chondrocytes, (iii) to illustrate the utility of this model by simulating the dependence of the chondrocyte resting membrane potential on identified electrolytes and osmolarity in synovial fluid (iv) to put our findings in the context of depolarization-secretion coupling in the chondrocyte based on data from recordings of TRP channel-mediated cation (Na^+^ and Ca^2+^) influx in chondrocytes (cf. Lewis et al., 2013; O'Conor et al., 2014).

## Methods

Mammalian chondrocytes express a number of different voltage- and ligand-gated ion channels, together with ion-selective pumps and exchangers as well as intercellular coupling proteins (cf. Barrett-Jolley et al., 2010; Asmar et al., 2016). In this study, we have extended this published data set using two different experimental preparations for recordings of ion selective currents in unstimulated chondrocytes. We have also complemented and extended these findings with the development of a mathematical model to account for regulation of the resting membrane potential, E_m_, in human chondrocytes. The new data sets presented in this paper are based mainly on patch clamp experiments which were done using enzymatically isolated individual human chondrocytes obtained from a knee replacement program (The Southern Alberta Transplant Service). These cells, held in 2D culture for 1–3 days, were not passaged and are therefore classified as “primary.”

### Experimental conditions

In the experimental conditions employed in this study, isolated human chondrocytes had E_m_ values ranging from −30 to −60 mV when superfused with normal Tyrode's solution and studied using standard whole-cell patch clamp methods (Clark et al., 2011). This range of resting membrane potential values may reflect the intrinsic heterogeneous physiological states of these cells. However, as we have reported previously, some of this variability is very likely to result from the fact that in these very small, approximately spherical cells (diameter, ~7 microns; capacitance, ~6–12 pF), the patch pipette recording method is being applied very near its maximal technical capabilities (Wilson et al., 2011). That is, the input resistance of the chondrocyte is very large (5–10 GΩ), and the maximum seal resistance between the surface membrane of the chondrocytes and the polished surface of the glass pipette is comparable to 5–15 GΩ. The consequence is that the actual chondrocyte membrane potential may be underestimated due to the current flow through the seal resistance. In most circumstances this results in a depolarization, as noted in our previous work (Wilson et al., 2011).

### Electrophysiological studies

For these electrophysiological studies, selected populations of chondrocytes were first plated on pieces of glass coverslips, which were then transferred from the culture dishes to our superfusion chamber at the start of each experiment. Only single isolated cells with a smooth surface rounded appearance were selected for these recordings using standard patch-clamp methods (Clark et al., 2011).

Patch pipettes were fabricated from non-heparinized hematocrit capillaries. Patch pipette-filling solutions were either (i) K^+^-rich (KCl) or (ii) Cs^+^-rich (CsCl), depending on the protocol. In most experiments, free Ca^2+^ concentration in the pipette solutions was buffered to very low levels (<10 nM) by 10 mM EGTA, without added Ca^2+^. The D.C. resistance of the pipettes when filled with internal solutions was in the range ~2–4 MΩ. The seal resistance before breaking into the chondrocyte to begin whole-cell recording ranged from 4.8 to 72.3 GΩ (mean ± s.e.m.; 16.1 ± 1.5 GΩ, *n* = 66). Successful seals formed very rapidly (~1–2 s), and the subsequent break-in to the cells for whole-cell recording was “clean”; access resistance was generally about twice the value of the pipette resistance. (cf. Clark et al., 2011).

All electrophysiological measurements were made with a Multi-Clamp 700 A patch clamp amplifier (Molecular Devices). Membrane currents and potentials were digitized with a 1,322 A data acquisition system, stored on a microcomputer and analyzed off-line with PClamp (version 8). The “standard” voltage-clamp protocols consisted of (i) 1 s voltage ramp from −100 to +100 mV (holding potential generally −80 mV), repeated at a frequency of 0.2–0.5 Hz, and (ii) a “step” protocol, consisting of 500 ms steps from a holding potential of −80 mV membrane to potentials between −100 and +100 mV. In some experiments, a “P/n” protocol was used to correct “step” currents for linear leakage and capacity transient currents. Since the ramp and step protocols gave very similar current-voltage (I–V) relationships, the ramp protocol was used to obtain rapid, repetitive measurement of the I–V, e.g., during drug applications.

Transmembrane current values were normalized to cell capacitance, which was measured from the area under the capacitative current transient produced by a +5 mV step in membrane potential. Capacitance was recorded before and after break-in to the cell; the capacitance of each single chondrocyte was taken as the difference in these capacitance values. Drugs were delivered to cells with a multi-barreled local superfusion device that changed the solution around a cell within <1 s. All experiments were carried out at room temperature (20–22°C).

In the second experimental part of this study, after obtaining the data that characterized the predominant K^+^ currents in enzymatically isolated human chondrocytes, we analyzed TRP channel mediated currents (**Figures 8**, **9**) using a different source human articular chondrocytes. These cells were from a chondrocyte preparation made available by Glaxo Smith Kline Ltd (GSK) (Balakrishna et al., 2014). GSK cells that were cultured from frozen samples of primary cells (batches #1060, #1274). The culture medium was DMEM/F12, supplemented with 10% fetal calf serum, 2 mM L-glutamine and penicillin/streptomycin (1:10). These chondrocytes were used up to a maximum of 6 days after plating and were not passaged. TRP channels were activated or blocked using proprietary compounds that were obtained from Glaxo Smith Kline; GSK Ltd. (Thorneloe et al., 2008; Hilfiker et al., 2013).

### The atypical microenvironment of the chondrocyte

In adult mammals, the chondrocyte cell population is in a physiological environment, the articular joint fluid that differs significantly from that of most other cells in healthy human tissues. A number of these important differences are listed in Table 1. Note that the extracellular fluid within the articular joint is hypertonic (~320 mOsm vs. blood plasma, ~280 mOsm). In addition, the extracellular pH, i.e., that of the synovial fluid that bathes the chondrocyte is somewhat acidic, pH 7.2; and the extracellular [K^+^]_o_ levels are significantly elevated measuring ~10–15 mM, as opposed to 4.0–5.4 mM [K^+^]_o_ in normal mammalian plasma (Huber et al., 2000; Wilkins et al., 2000a). Nevertheless, the main large transmembrane electrochemical gradients for Na^+^, K^+^, and Cl^−^ in chondrocytes are quite similar to those in other mammalian cells. Establishment and maintenance of these gradients leads to the requirement for an ATP-dependent Na^+^/K^+^ pump mechanism (assumed to be electrogenic). This maintains ionic homeostasis and stabilizes cell volume. Evidence for expression of a Na^+^/K^+^ ATPase has been obtained in bovine articular chondrocytes (Mobasheri et al., 1997). It is also known that glucose is the major energy substrate for articular chondrocytes and that these cells express Glut1 and Glut3 glucose transporter (Phillips et al., 2005). The Mobasheri Group (Mobasheri et al., 2008) have also reported that the expression, distribution and function of these facilitative glucose transporters are regulated significantly by hypoxia, inflammation, or altered complements of articular joint growth factors.

**Table 1:** Ion concentrations in compartments within the mammalian knee joint (see Ref. Hall et al., 1996) (adapted from Table 1 in Wilkins et al., 2000a).

**Electrolyte concentrations (mM)**	**Cytoplasm**	**Matrix**	**Sinovial fluid**
[Na^+^]	40	240–350	140
[K^+^]	120–140	7–12	5
[Ca^2+^]	1-5 × 10^−5^	6–15	1.5
[Cl^−^]	60–90	60–100	140
[${\text{HCO}}_{3}^{-}$]	20	15	23
[${\text{SO}}_{4}^{2-}$]	0.17	0.30	0.81
pH	7.1–7.2	6.6–6.9	7.4
Osmolarity (mOsm)	–	350–450	300

The fixed negative charges on proteoglycans of the extracellular matrix are in the immediate vicinity of the chondrocyte and can attract cations (e.g., Na^+^), while also excluding anions. As a result, localized cation accumulation occurs (Table 1), and for this reason there is a significant osmotically driven water influx (Urban et al., 1993; Wilkins et al., 2000a; Lewis et al., 2011). In addition, intrinsic characteristics of the “pericellular matrix” on the immediate surface of each chondrocyte can serve as a significant diffusion barrier, as demonstrated recently with the use of optical trap methods (McLane et al., 2013). It is also important to note that the literature now suggests that the relevant functional unit of the chondrocyte is the chondron. It includes the cell (chondrocyte) and its glycocalyx or pericellular coat (Muir, 1995; Poole, 1997; Guilak et al., 2006).

The articular joint receives only very limited blood supply. Accordingly, the synovial fluid must supply adult articular cartilage with the required (small amounts of) nutrients, as well as sufficient oxygen to maintain Na^+^/K^+^ pump activity and ensure intracellular Ca^2+^, [Ca^2+^]_i_, homeostasis (Mobasheri et al., 1997; Mobasheri, 1998; Chao et al., 2006). Metabolic byproducts are removed mainly by diffusion (Urban et al., 1993; Wilkins et al., 2000b). An important consequence of the “avascular” nature of this articular joint tissue is that chondrocytes generate ATP by substrate-level phosphorylation during anaerobic respiration (Guilak et al., 1997). This generates H^+^ ions as a byproduct, and this tends to acidify the pH in this micro-environment. The dynamic changes in mechanical loading within the knee joint during activity also expose chondrocytes to very significant fluctuations in vector and shear forces. The resulting mechanical changes can activate mechano-or shear-sensitive ion channels (Hall et al., 1996; Lane Smith et al., 2000; Mouw et al., 2006).

## Model development

**Figure 1:**
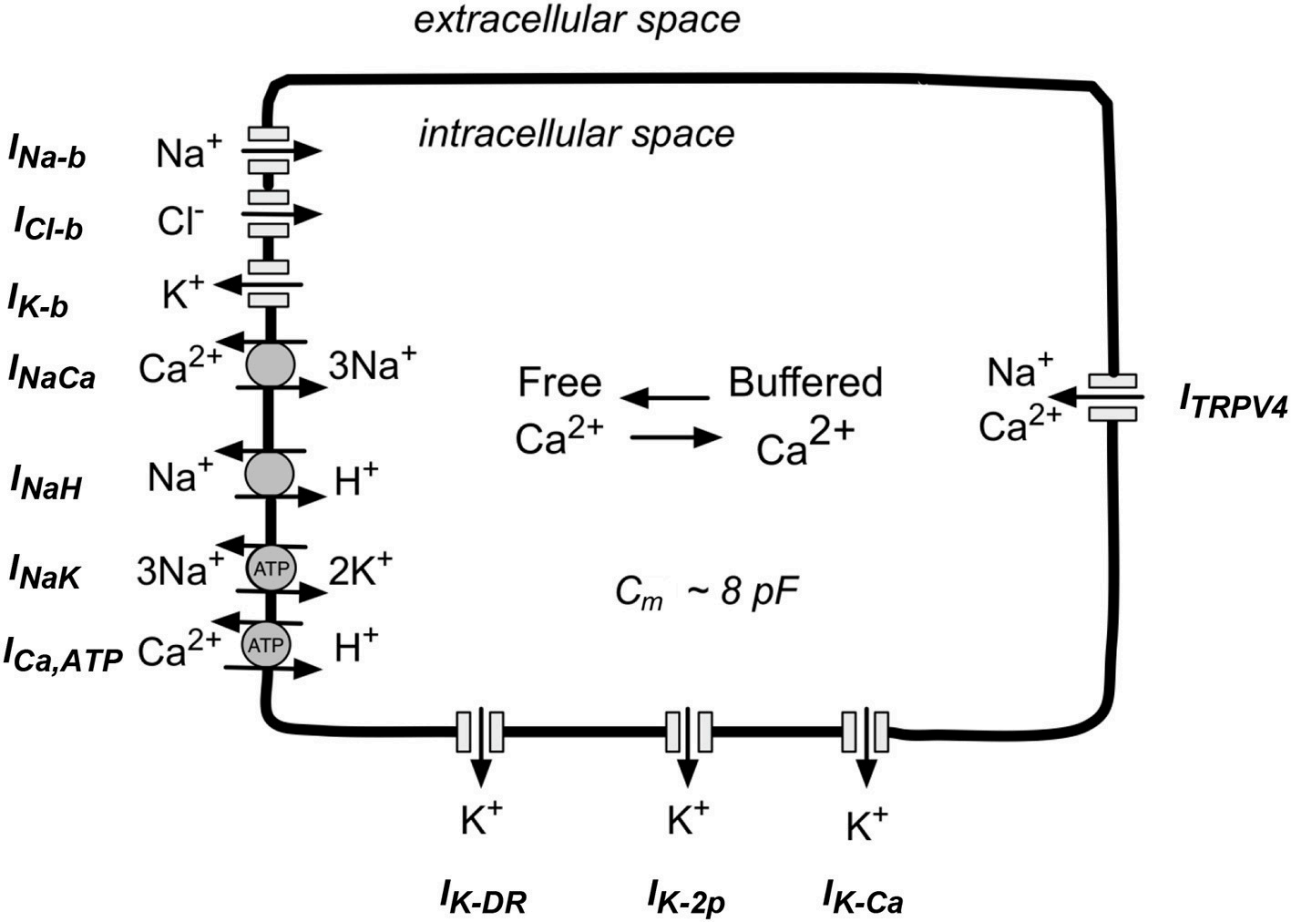
Schematic illustration of the main ion selective channels, ion exchange proteins, and ATP-dependent ion pumps that are known to be expressed in human chondrocytes. This information forms the basis for our mathematical model of chondrocyte K^+^ transport and resting potential, E_m_. The three ion selective channels labeled at the top left of this diagram are so-called background channels; these currents show no time dependence. The three types of ion channels shown on the bottom are the focus of this paper. These K^+^-selective channels in human chondrocytes, have been studied in detail in our Laboratory and by other groups (see section Results). The ion-selective pumps and exchangers shown at the bottom left are necessary to maintain volume, and contribute to electrolyte homeostasis in the human chondrocyte. The TRPV4 channels shown on the right can allow Na^+^ and Ca^2^+ influx. In addition, (see section Discussion) chondrocytes can exhibit cell-to-cell coupling via connexin-mediated ion and metabolite transfer, and/or “hemichannel behavior” (see section Discussion).

We have developed a new, first generation mathematical model for the resting membrane potential, E_m_, of the chondrocyte, based partly on the experimental data obtained and described in section Electrophysiological Studies. This includes (a) K^+^ currents, (b) time-independent currents, (c) pump and exchanger currents, and (d) intracellular Ca^2+^ buffering. These components are illustrated in Figure 1 and are described in detail in following sections. The transmembrane ion transport processes include: *I*_*K*−*DR*_, a voltage-dependent delayed rectifier K^+^ channel; *I*_*K*−*Ca*_, voltage and internal Ca^2+^-dependent K^+^ channel; *I*_*K*−2*p*_, a two-pore K^+^ channel; *I*_*K*−*ATP*_, ATP-dependent K^+^ channel; *I*_*Na,b*_, a time-independent “background” Na^+^ channel; *I*_*K,b*_, a time-independent “background” K^+^ channel; *I*_*Cl,b*_, a time-independent “background” Cl^−^ channel; *I*_*NaK*_, ATP-dependent electrogenic Na^+^-K^+^ pump; *I*_*NaCa*_, electrogenic Na^+^-Ca^2+^ exchanger; *I*_*NaH*_, the transmembrane Na^+^ flux of the electroneutral Na^+^-H^+^ antiporter; *I*_*Ca,ATP*_, electroneutral ATP-dependent Ca pump; and *I*_*TRPV*4_, cation permeable TRPV4 ion channel. In this model, intracellular Ca^2+^ is buffered by binding to calmodulin.

The equation governing the transmembrane potential, V, of the chondrocyte is

(1) $$\begin{array}{l} C_{m}\frac{dV}{dt}=\;-(I_{K-DR}+I_{K-Ca}+I_{K-2p}+I_{K-ATP}+I_{Na,b}+I_{K,b}\\ \;+I_{Cl,b}+I_{NaK}+I_{NaCa}+I_{TRPV4}) \end{array}$$

where *C*_m_ is chondrocyte capacitance (8 pF). This equation includes all transport processes that are electrogenic i.e., generate net transmembrane current(s).

The intracellular concentrations of Na^+^, [Na^+^]_i_ and K^+^, [K^+^]_i_, are governed by two transport equations, namely,

(2) $$\begin{array}{l} \frac{d{\left[Na^{+}\right]}_{i}}{dt}=\;\frac{-\left(I_{Na,b}+3I_{NaK}+{3I}_{NaCa}-I_{NaH}\right)}{vol_{i}\;F} \end{array}$$

and

(3) $$\begin{array}{l} \frac{d{\left[K^{+}\right]}_{i}}{dt}=\frac{-\left(I_{K,b}-2I_{NaK}+I_{K-DR}+I_{K-2p}+I_{K-Ca}+I_{K-ATP}\right)}{vol_{i}\;F} \end{array}$$

where *vol*_*i*_ is the internal volume of the chondrocyte (calculated to be 0.005884 mL), and *F* is the Faraday constant, 96,485 C/mol. Note that Equation (1) does not include *I*_*NaH*_, since this is the Na^+^ flux generated by the electroneutral Na^+^-H^+^ antiporter, but this term is included in the Na^+^ homeostasis Equation (2). Equations (2) and (3) specify how the intracellular concentrations of Na^+^ and K^+^ evolve with time, and also how the Nernst potentials for Na^+^, and for K^+^, change with time. *R* = 8.314 J K^−1^ mol^−1^ is the universal gas constant, *T* = 310.15 is body temperature in deg. Kelvin, and *z*_*Na*_ = 1 and *z*_*K*_ = 1 are ionic valances for Na^+^ and K^+^, respectively. The extracellular concentrations of Na^+^, [Na^+^]_o_, and K^+^, [K^+^]_o_, are held constant during each simulation. However, some simulations were done after changing these values to those that are more representative of the physiological milieu of the chondrocyte, (shown in Table 1), rather than those set by our experimental superfusate for the patch-clamp experiments.

Intracellular Ca^2+^ concentration, [Ca^2+^]_i_, also changes with time, due to transmembrane transport of Ca^2+^ by the Na^+^-Ca^2+^ exchanger and ATP-dependent Ca^2+^ pump, and intracellular calmodulin binding, according to the equation:

(4) $$\begin{array}{l} \frac{d{\left[Ca^{2+}\right]}_{i}}{dt}\;=\;\frac{\left(I_{NaCa}-I_{Ca,\;ATP}\right)}{vol_{i}\;F}-0.045\;\frac{dO_{c}}{dt} \end{array}$$

where ***O***_*c*_ is the fraction of intracellular calmodulin bound to Ca^2+^ (see Equation 39).

Differential equations (1–4) and those for *I*_K,Ca_ and calmodulin buffering were solved numerically. All data simulations and processing was performed off-line using both noncommercial and commercial software packages (Radhakrishnan and Hindmarsh, 1993; Maleckar et al., 2009; Eaton et al., 2014), as well as custom, in-house scripts. The software that is the basis of our model of the chondrocyte resting membrane potential is available on request.

## Results

### Ion-selective transmembrane currents

#### Potassium currents

We have identified and partially characterized three different K^+^ currents in mammalian chondrocytes. In principle, each of these can contribute to the resting membrane potential, E_m_, either at baseline or following selective activation/enhancement by physiological stimuli or pathophysiological conditions. These K^+^ currents are: (i) a time-and voltage dependent delayed rectifier K^+^ current, *I*_*K*−*DR*_; (ii) a K^+^ current due to 2-pore K^+^ channels, *I*_*K*−2*p*_; and (iii) a large conductance voltage and Ca^2+^-activated K^+^ current, *I*_*K*−*Ca*_, have been studied in some detail in our laboratory (Wilson et al., 2004; Clark et al., 2010, 2011, respectively) and also by other groups (for reviews, see Grandolfo et al., 1992; Mobasheri et al., 2007, 2012; Barrett-Jolley et al., 2010; Asmar et al., 2016). An ATP-dependent K^+^ current (*I*_*K*−*ATP*_) has also been identified in patch clamp studies published by other investigators (cf. Mobasheri et al., 2007).

**Figure 2:**
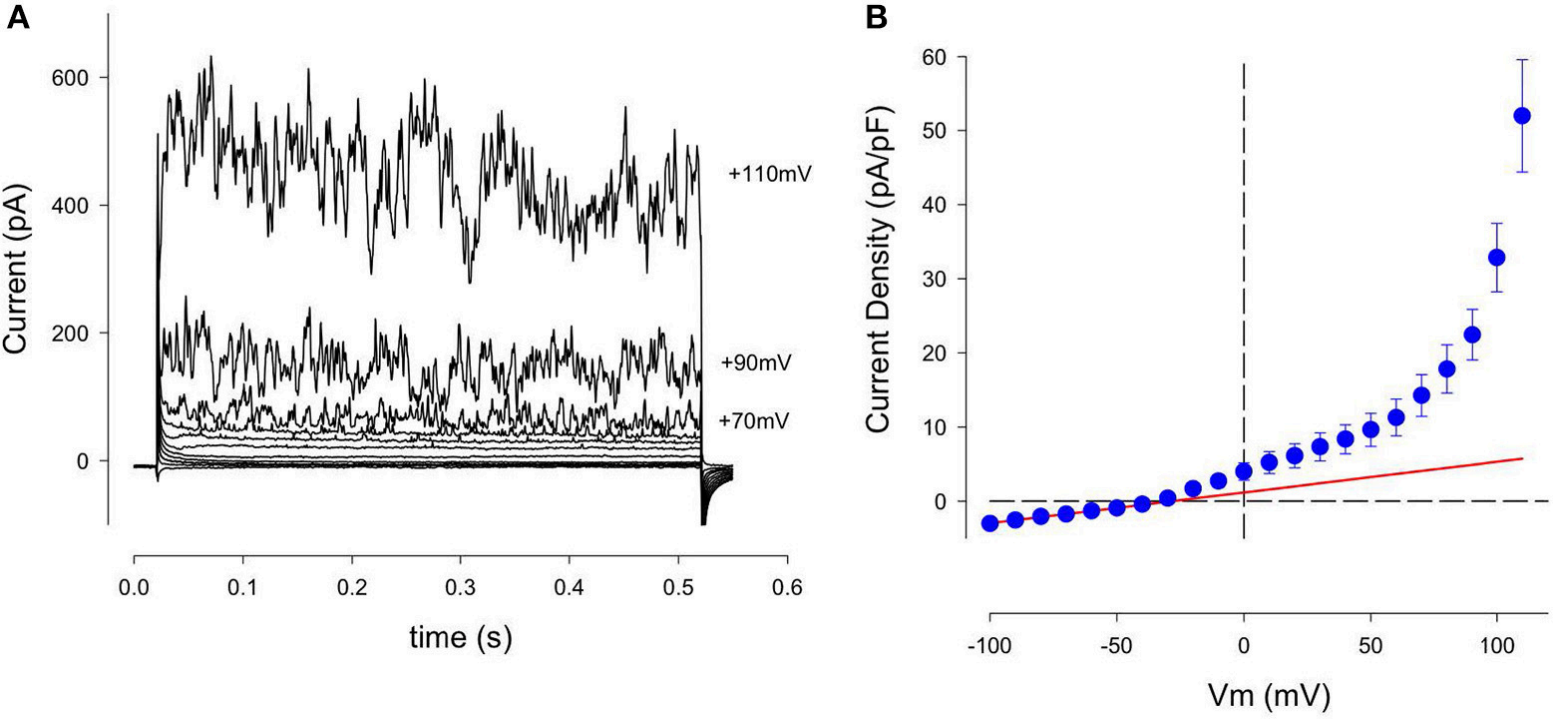
Family of K^+^ currents recorded from an isolated human chondrocyte maintained in primary 2D culture conditions. **(A)** These currents were recorded from a chondrocyte that was held at −80 mV, and then stepped to membrane potentials between −100 and +110 mV, in 10 mV increments. Current records are shown between −90 and +100 mV, (in 20 mV increments). This chondrocyte had been in culture for 3 days; its capacitance, C_m_, was 7.3 pF. Panel **(B)** is an averaged current-voltage (I–V) relation for 13 cells, (1 day in culture). Currents recorded from each cell were normalized to cell capacitance before averaging. Solid red trace is the best-fit straight line between −100 and −50 mV. Its slope is 0.042 nS/pF. Mean ± s.e.m. C_m_ of 13 cells was 7.4 ± 0.8 pF.

A typical pattern of K^+^ currents from our patch clamp recordings using single isolated human articular chondrocytes (denoted HAC) is illustrated in Figure 2A. This chondrocyte first held at −80 mV, and then the membrane potential was stepped to selected levels between −100 and +110 mV, in 10 mV increments at a rate of 0.2 Hz. Three consistent features are noteworthy: (i) over much of this membrane potential range, the currents showed no obvious time- and voltage dependence, (ii) after being activated, these currents became much greater in amplitude and showed larger fluctuations with increasingly depolarized membrane voltages; (iii) large, very noisy outward currents began to appear at about +60 mV.

Figure 2B consists of an averaged isochronal current-voltage (I–V) relationship based on data obtained from 13 human chondrocytes, each studied after 1 day in cell culture. The magnitude of these K^+^ currents was normalized to the capacitance of each cell in order to compensate for differences in cell size. Note that this I–V relationship is approximately linear at membrane potentials negative to about −50 mV but becomes non-linear at more depolarized voltages. The solid red trace shows the best-fit straight line for the data between −50 and −100 mV, extrapolated over the entire potential range of the I–V plot. The nonlinearity at depolarized membrane potentials is mainly due to increases in the noisy outward current. This feature is very evident at membrane potentials positive to about +60 mV. Note also that there is a small non-linearity in the I–V between ~−40 and +50 mV. This suggests that another component of outward current might be present in these single HAC preparations.

##### Delayed rectifier K^+^ current: I_K-DR_

A conventional time- and voltage-dependent delayed rectifier K^+^ current has been identified in mouse, canine, rabbit, and human articular chondrocytes under primary culture conditions (Wilson et al., 2004; Barrett-Jolley et al., 2010; Clark et al., 2010). Our previous analysis of ion selectivity, as judged by reversal potential measurements, has shown that this current, denoted *I*_*K*−*DR*_, is carried mainly by K^+^ (Wilson et al., 2004; Clark et al., 2010). Moreover, the biophysical properties of this current and its pharmacological blockade suggested that it is generated by a well-known Kv1.x conductance family, perhaps Kv1.5 or Kv1.6.

**Figure 3:**
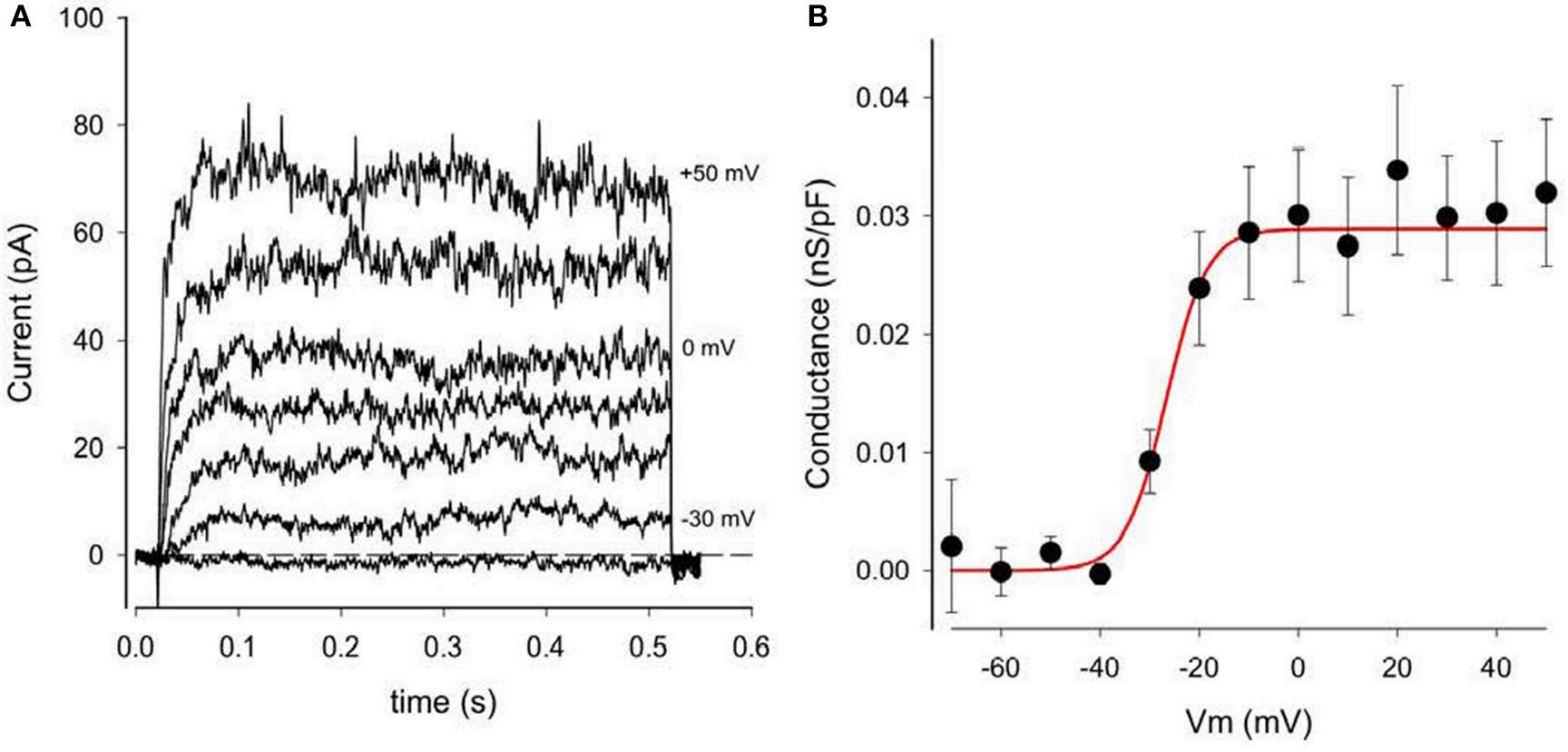
Delayed rectifier K^+^ currents, *I*_K−DR_, in a human chondrocyte. **(A)** Examples of I_K−DR_ activated by voltage-clamp steps from holding potential of −80 mV to membrane potentials of −40, −30, −20, −10, 0, +20, and +50 mV. Records have been corrected for capacity and linear leak currents. **(B)** Plot of I_K−DR_ peak conductance (per pF) vs. membrane voltage, V. Conductance was obtained by dividing peak I_K−DR_ by (V-E_K_), where E_K_ is K^+^ Nernst potential (−83 mV). Data from 16 cells (mean ± SEM). The best-fit Boltzmann relationship (red line) is the factor αK-DR (Equation 2).

The presence of this small time- and voltage-dependent current in these human chondrocyte (HAC) preparations was revealed more clearly when the linear leak and capacity currents were removed from the raw current records (see Methods section). An example of current records from one HAC preparation in which these correction procedures were used is shown in Figure 3. The records in Figure 3A clearly reveal the time- and voltage-dependent properties of the relatively small component of outward current that is activated at membrane potentials positive to ~-40 mV. The rate of activation of this current increases significantly with progressively larger depolarizations. Note, however, these K^+^ currents showed no inactivation during the 500 ms voltage-clamp steps. In summary, the properties of this current resembled the “delayed rectifier” K^+^ current, *I*_*K*−*DR*_ that we and others have reported previously from studies in chondrocytes from other species (Wilson et al., 2004; Clark et al., 2010). Not all isolated human chondrocytes expressed this type of “delayed-rectifier” K^+^ current. As an example, in one group of 13 HAC studied after 1 day in culture, only 8 cells expressed a detectable *I*_*K*−*DR*_. In a second group of 12 HAC studied after 3 days in culture, only 4 cells expressed *I*_*K*−*DR*_. These findings suggest that there is intrinsic variability in the expression of this K^+^ current; and/or that its expression may decrease with time in culture (see section Discussion). Figure 3B shows a plot of *I*_*K*−*DR*_ conductance vs. membrane voltage, E_m_, for 16 cells. The *I*_*K*−*DR*_ conductance values were obtained by dividing peak *I*_*K*−*DR*_ by the electrochemical driving force. (EMF), which is defined as the membrane potential, V, minus the Nernst potential for K^+^, E_k_ (~−83 mV under the experimental recording conditions). The best-fit Boltzmann relationship (red line) in Figure 3B yields a descriptor of the voltage dependent activation of this K^+^ current. *I*_*K*−*DR*_ was described by the following expression, as we have published previously (Maleckar et al., 2009);

(5) $$\begin{array}{l} I_{K-DR}\;=g_{K-DR}\;\alpha_{KDr}\left(\text{V}-{\text{E}}_{\text{K}}\right) \end{array}$$

where

g_*K*−*DR*_ is the maximal *I*_*K*−*DR*_ conductance, namely 0.0289 nS/pF from Figure 3B, and

α_*KDr*_ is a voltage-dependent activation factor, given by the expression

(6) $$\begin{array}{l} \alpha_{KDr\;}=\;\frac{1.0}{(1.0+\;e^{\frac{-(V+26.7)}{4.1}}} \end{array}$$

Note that the extracellular potassium concentration, [K^+^]_o_, was initially set to 5.4 mM under “typical” physiological conditions. In fact, however, [K^+^]_o_ is likely to be in the 5–15 mM range in the microenvironment of the chondrocyte *in situ* (see Table 1). Intracellular K^+^ concentration was initially set to 140 mM in this parameterization of the model, but evolves through time, governed by Equation (3).

##### Ca^2+^-activated K^+^ current: *I*_*K*−*Ca*_

The prominent fluctuations in outward current traces recorded from all human chondrocytes at strongly depolarized membrane potentials suggested the expression of this “large” variant of Ca^2+^-activated K^+^ channels (“BK,” KCa1.1). It is well-known that the voltage dependent gating of these BK channels is strongly modulated by the intracellular Ca^2+^ concentration, [Ca^2+^]_i_ (cf. Horrigan and Aldrich, 2002; Magleby, 2003; Berkefeld et al., 2006, 2010; Barrett-Jolley et al., 2010; Mobasheri et al., 2012; Asmar et al., 2016). In the experimental results in Figure 2 [Ca^2+^]_i_ was held very low (<10 nM) by using a patch pipette solution containing no added Ca^2+^, combined with a strong Ca^2+^ buffer (10 mM EGTA). In the presence of this very low [Ca^2+^]_i_, these BK channels can be activated only by very strong voltage clamp depolarizations (e.g., >+40 mV).

**Figure 4:**
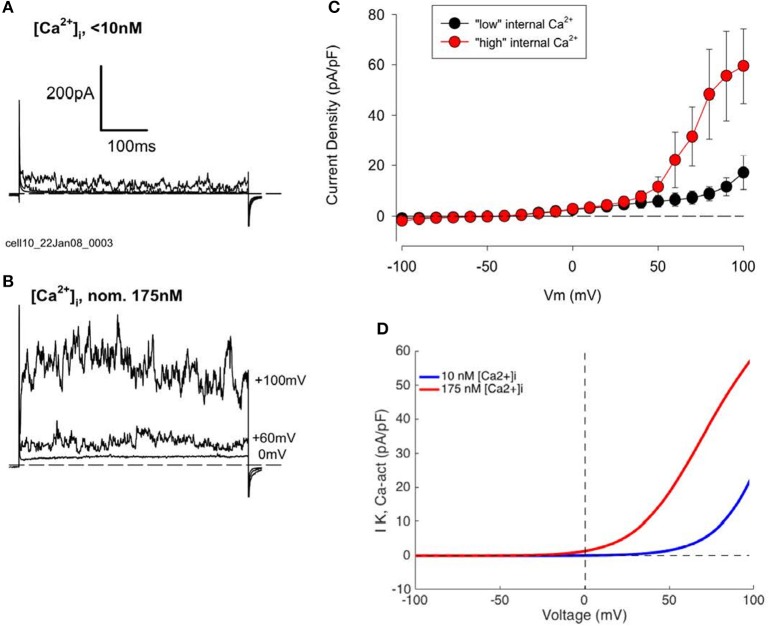
Effect of changes in intracellular Ca^2+^, [Ca^2+^]_i_, concentration on K^+^ currents in human articular chondrocytes. **(A)** Example of currents from a HAC with “low” internal [Ca^2+^]_i_ (0 [Ca^2+^]_i_, 10 mM EGTA). Currents produced by voltage steps to 0, +60, and +100 mV from a holding potential of −80 mV are shown. C_m_ = 9.72 pF. **(B)** Currents from a cell with “high” internal [Ca^2+^]_i_ (3 mM added Ca^2+^, 10 mM EGTA; nominal Ca^2+^ concentration = 175 nM). Voltage-clamp steps were to 0, +60, and +100 mV. C_m_ = 6.24 pF. Currents in **(A,B)** were not leak or capacity current corrected. **(C)** I–V relations for groups of HAC with “low” (n = 5, blue) and “high” (n = 4, red) [Ca^2+^]_i_ patch-clamp solution. These chondrocytes were all from same batch, 1 day in culture. **(D)** Model I–Vs for I_K−Ca_, with different internal [Ca^2+^] levels (from Equation 4).

To more clearly reveal the functionally important properties of this BK current, additional experiments were carried out using a pipette solution containing 3 mM Ca^2+^ and 10 mM EGTA, which yielded a nominal free Ca^2+^ concentration of ~175 nM. Data from these experiments are summarized in Figure 4. Currents from two groups of HAC's, one with “low” internal Ca^2+^ solution, and one with “high” Ca^2+^, are shown in Figures 4A,B, respectively. It is clear that the “noisy” outward currents were much larger in the presence of “high” [Ca^2+^]_i_ compared with “low.” Figure 4C compares pooled I–V data from subsets of HAC preparations perfused internally with either “low” or “high” internal [Ca^2+^]_i_. All of these chondrocytes were from the same batch, and recordings were made after 1 day in conventional 2D cell culture. As expected for currents generated by BK channels the large, fluctuating outward currents recorded from chondrocytes with “high” [Ca^2+^]_i_ were activated at considerably more negative membrane potentials than from chondrocytes with “low” [Ca^2+^]_i_ (Horrigan and Aldrich, 2002; Berkefeld et al., 2010). Moreover, the maximum current (measured at +100 mV) was several times larger in the “high” [Ca^2+^]_i_ chondrocytes. These results strongly suggest that a Ca^2+^-activated current I_K−Ca_, produced by the so-called BK channels, is an important component of K^+^ current in these cultured HAC cells (Magleby, 2003; cf. Sun et al., 2009).

It is well-known that the molecular properties and biophysical characteristics of the extensive Ca^2+^ activated K^+^ channel family can be used to divide them into three sub-groups (Berkefeld et al., 2010). Defining characteristics include: (i) the specific biophysical properties of the current (e.g., its voltage dependence), (ii) their pharmacological profile (e.g., sensitivity to block by apamin or tetraethylammonium, TEA), or (iii) the single channel conductance. In the case of human chondrocytes (as shown in Figures 2, 3) the pronounced current fluctuations (noise) strongly suggest the presence of the variant of Ca^2+^ activated K^+^ channels known as the large conductance subtype, BK. The major properties of this current (cf. Horrigan and Aldrich, 2002) have been incorporated into a detailed mathematical model developed by Sun et al. (2009).

The mathematical description for this Ca^2+^-activated K^+^ current is a 10-state kinetic Markov-type model, including four calcium-binding steps, with all the voltage dependence assigned to the transitions between closed and open states, i.e., the C-O equilibrium:

(7) $$\begin{array}{l} \frac{dC_{O}}{dt}=\beta_{O}O_{o}-\;\alpha_{O}C_{o}+K_{c}C_{1}-{4CaC}_{o} \end{array}$$

(8) $$\begin{array}{l} \frac{dC_{1}}{dt}=\beta_{1}O_{1}-\;\alpha_{1}C_{1}-K_{c}C_{1}+{4CaC}_{o}-{3CaC}_{1}+{2K_{c}C}_{2} \end{array}$$

(9) $$\begin{array}{l} \frac{dC_{2}}{dt}=\beta_{2}O_{2}-\;\alpha_{2}C_{2}-{2K}_{c}C_{2}+{3CaC}_{1}-{2CaC}_{2}+{3K_{c}C}_{3} \end{array}$$

(10) $$\begin{array}{l} \frac{dC_{3}}{dt}=\beta_{3}O_{3}-\;\alpha_{3}C_{3}-{3K}_{c}C_{3}+{2CaC}_{2}-{CaC}_{3}+{4K_{c}C}_{4} \end{array}$$

(11) $$\begin{array}{l} \frac{dC_{4}}{dt}=\beta_{4}O_{4}-\;\alpha_{4}C_{4}-{4K}_{c}C_{4}+{CaC}_{3} \end{array}$$

(12) $$\begin{array}{l} \frac{dO_{0}}{dt}={-\beta }_{0}O_{0}+\;\alpha_{0}C_{0}+K_{0}O_{1}-4{CaO}_{0} \end{array}$$

(13) $$\begin{array}{l} \frac{dO_{1}}{dt}={-\beta }_{1}O_{1}+\;\alpha_{1}C_{1}-K_{o}O_{1}+{4CaO}_{o}-{3CaO}_{1}+{2K_{o}O}_{2} \end{array}$$

(14) $$\begin{array}{l} \frac{dO_{1}}{dt}={-\beta }_{2}O_{2}+\;\alpha_{2}C_{2}-{2K}_{o}O_{2}+{3CaO}_{1}-{2CaO}_{2}+{2K_{o}O}_{3} \end{array}$$

(15) $$\begin{array}{l} \frac{dO_{3}}{dt}={-\beta }_{3}O_{3}+\;\alpha_{3}C_{3}-{3K}_{o}O_{3}+{2CaO}_{2}-{CaO}_{3}+{4K_{o}O}_{4} \end{array}$$

(16) $$\begin{array}{l} \frac{dO_{4}}{dt}={-\beta }_{4}O_{4}+\;\alpha_{4}C_{4}-{4K}_{o}O_{4}+{CaO}_{3} \end{array}$$

where ***C***_n = 0,1,2,3,4_ and ***O***_n = 0,1,2,3,4_ are closed and open states 1 through 4, respectively, with the total open probability corresponding to the sum of the open states, **O;** α_n = 0,1,2,3,4_ represent rates corresponding to transition from a closed to an open state and β_n = 0,1,2,3,4_ represent rates corresponding to transition from an open to a closed state; K_o_ and K_c_ are off-rates from open and closed states, respectively, and *Ca* is the calcium on-rate, where

(17) $$\begin{array}{l} \alpha_{n\;}=\;{A_{n}\;e}^{\frac{z_{CO}VF}{RT}} \end{array}$$

(18) $$\begin{array}{l} \beta_{n\;}=\;{B_{n}\;e}^{\frac{z_{OC}VF}{RT}} \end{array}$$

*A*_0_ = 0.659, *A*_1_ = 3.955, *A*_2_ = 25.05, *A*_3_ = 129.2, *A*_4_ = 261.1;
*B*_0_ = 2651.7, *B*_1_ = 1767.8, *B*_2_ = 1244.0, *B*_3_ = 713.0, *B*_4_ = 160.0;
*z*_*CO*_ = 0.718, *z*_*OC*_ = 0.646, *K*_*c*_ = 13.5, *K*_*O*_ = 1.5,

and the Ca^2+^ on-rates per site are 10^9^ M^−1^s^−1^, Ca^2+^ off-rates from *C*_n_ per binding site are 10^9^ *K*_*C*_ (13,500 s^−1^) and Ca^2+^ off-rates from *O*_n_ per binding site are 10^9^ *K*_*O*_ (1,500 s^−1^), and *R* is the universal gas constant, = 8.314 J K^−1^ mol^−1^ and *T* = 310.15 is body temperature in Kelvin.

*I*_*K*−*Ca*_ is then defined by:

(19) $$\begin{array}{l} I_{K-Ca}\;=\;{\text{g}}_{K-Ca}\text{O}\left(\text{V}-{\text{E}}_{\text{K}}\right) \end{array}$$

where g_*K*−*Ca*_ is the maximal conductance of the channel, equal to 2.50 nS/pF, **O** is the total open probability as given above, V is the transmembrane potential, and E_K_ is the Nernst potential for potassium.

We have used this mathematical formalism to compute I–V relationships for BK currents that can assumed to have been recorded from isolated human chondrocytes under conditions in which the composition of the pipette solution was adjusted (buffered) so that the [Ca^2+^]_i_ was ~10^−8^ M. In this situation, this K^+^ current can be activated at only very positive membrane potentials. In contrast, when [Ca^2+^]_i_ was increased to ~175 nM this Ca^2+^-activated K^+^ current activates at much less strongly depolarized membrane potentials, as shown by the computations summarized in Figure 4D.

**Figure 5:**
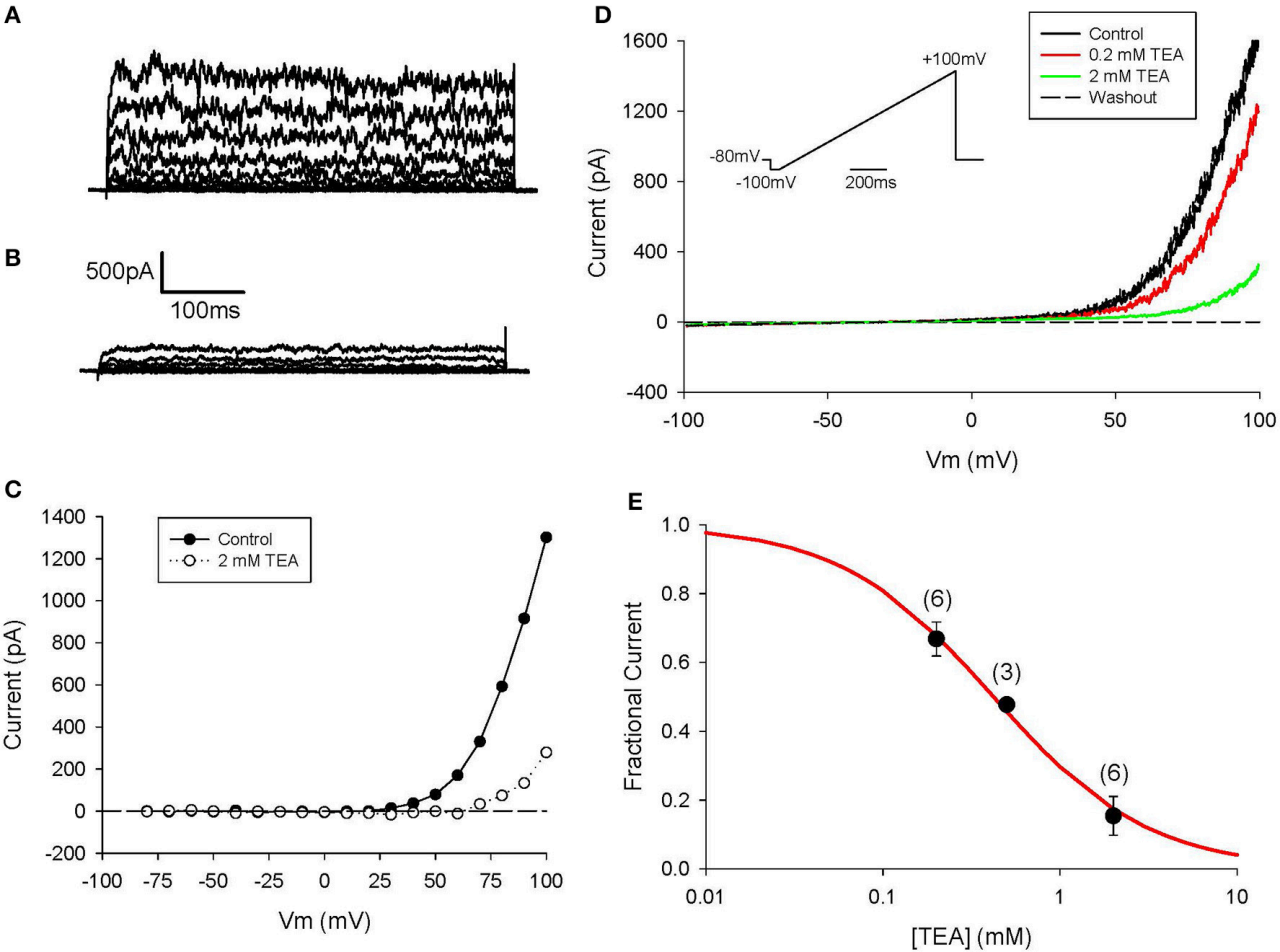
Block of I_K−Ca_ in human chondrocyte by TEA. **(A)** Control currents. The voltage-clamp protocol consisted of 500 ms steps from h.p. −80 to +100 mV. Linear leak and capacitive currents were removed using a P/3 protocol. **(B)** Currents in presence of 2 mM TEA. **(C)** I–V relations for control and TEA currents in **(A,B)**. **(D)** Currents in response to a ramp protocol (inset), in control and in presence of 0.2 and 2 mM TEA. Same cell as **(A,B)**. Cell capacitance was 12.0 pF; 3 days in culture. **(E)** Dose-response for TEA pooled from 3 to 6 cells (indicated above each data point). Each current amplitude was measured at +100 mV. Solid line is best-fit binding equation, with K_m_ = 0.42 mM.

Further information regarding the functional properties of this BK channel-mediated current was obtained using conventional pharmacological approaches. As shown in Figure 5 this K^+^ current can be blocked completely by concentrations of TEA that are known to quite selectively inhibit these BK channels. This pattern of results provides further evidence for the consistent and potentially prominent expression of this K^+^ current in human articular chondrocytes.

Although, *I*_*K*−*Ca*_ is a major outward current in human chondrocytes, it apparently does not contribute substantially to the resting potential under our conditions. This is because of the following: the input resistance of the human chondrocyte is very high (~5–10 GΩ); and under this circumstance, activation of a small number of these Ca^2+^ activated K^+^ channels would give rise to a resting potential that would be characterized by significant fluctuations in membrane voltage. Activation of these large conductance K^+^ channels is not consistent with recordings of resting membrane potential in the region of −40 mV (see Discussion section).

##### 2-Pore K^+^ current: *I*_*K*−2*P*_

Our previous work (Clark et al., 2011) defined recording conditions under which a K^+^ current generated by the TASK family of two-pore K^+^ channels could be identified consistently in single chondrocytes. This apparently very small current, that we denote *I*_*K*−2*p*_, exhibits no detectable time dependence (Goldstein et al., 2001). It is known that certain 2-pore K^+^ currents (including the TASK variants) are augmented by an increase in pH (alkalinization) of the extracellular medium (Patel and Honoré, 2001; Cid et al., 2013) and can be blocked by some local anesthetics (Kindler and Yost, 2005; Webb and Ghosh, 2009).

We have recorded this K^+^ current under high [K^+^]_o_ conditions, to ensure that the current changes are relatively large, so that their biophysical properties can be resolved. However, before these results can be put into a functional context, or incorporated into a mathematical model of human chondrocyte electrophysiology, they need to be corrected (scaled) to physiological conditions (i.e., normal [K^+^]_o_ levels). This can be done based on the Eisenman principle (cf. Hille, 2001): the conductance of an ion-selective channel scales according to the square root of the extracellular concentration of the permeant ion. Accordingly, *I*_*K*−2*p*_ is described by the classical Goldman-Hodgkin-Katz equation for a single ion species, with a square-root scaling factor to account for [K^+^]_o_:

(20) $$\begin{array}{l} I_{K-2p}=\frac{P_{k}z^{2}VF^{2}}{RT}\frac{\left({\left[K^{+}\right]}_{i}-{\left[K^{+}\right]}_{o}e^{\frac{-zVF}{RT}}\right)}{\left(1-e^{\frac{-zVF}{RT}}\right)} \end{array}$$

where *P*_*K*_ is a [K^+^]-dependent scaling factor that describes the permeability (conductance) for this K^+^ current, namely 3.1x10^−6^ √([K^+^]_i_/[K^+^]_o_), and z = 1 is the ionic valence for potassium.

**Figure 6:**
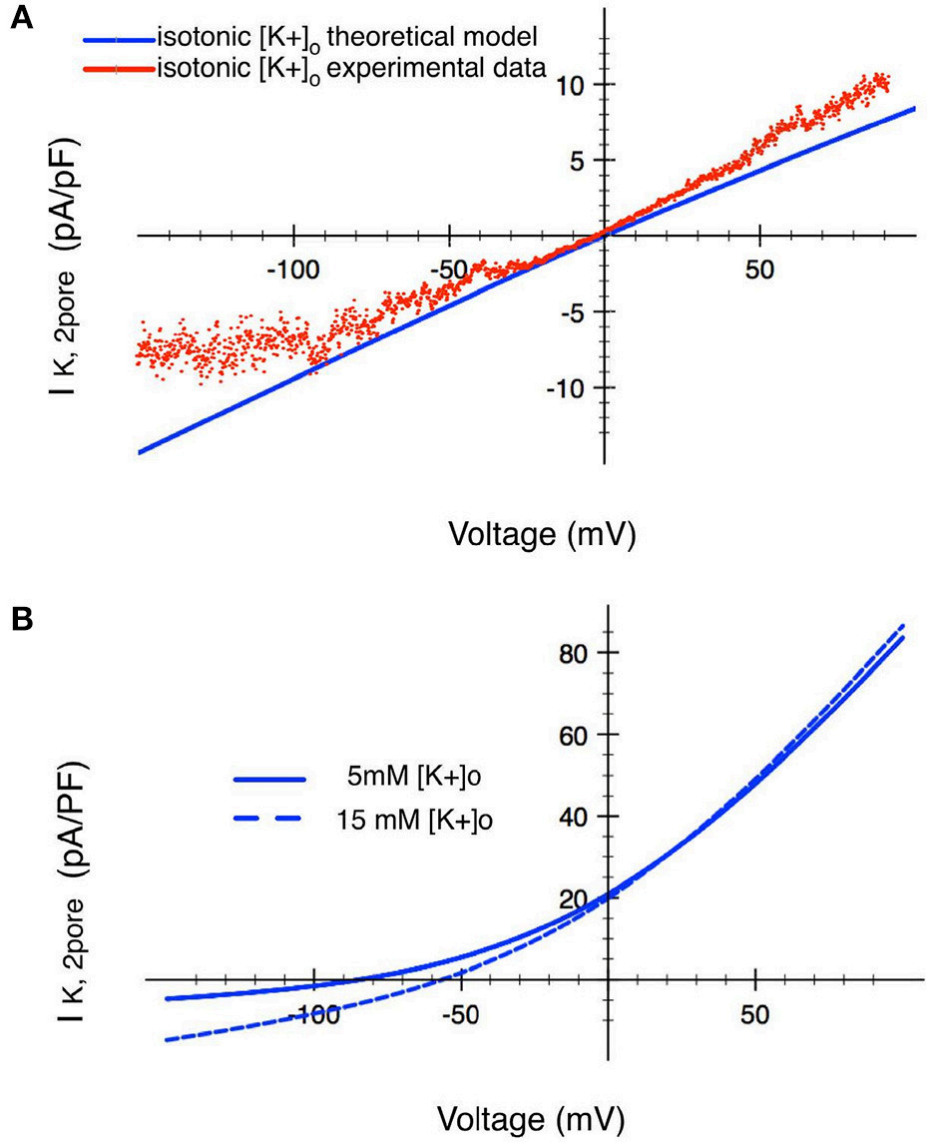
Analysis of the ion transfer function (I–V relationship) for a 2-pore K^+^ current in human chondrocytes. **(A)** Raw experimental data plotted as an I–V curve together with a superimposed I–V relationship (red trace) based on the mathematical formulation given in the text. These results were obtained in isotonic [K^+^]o conditions. The two traces in **(B)** show corresponding I–V relationships derived using the Eisenman Principle, so that this K^+^ current can be studied in conditions ([K^+^]o of 5 mM and 15 mM) that are in the physiological range. The blue trace shows the I–V relationship for this 2-pore K^+^ channel when [K^+^]o is 15 mM. The black trace shows the change in this ion transfer relationship when [K^+^]o is decreased to 5 mM. (see Table 1 and section Discussion).

The I–V relationship in Figure 6A shows that our primary data (Wilson et al., 2004) recorded in isotonic [K^+^] (~145 mM), has the expected reversal potential (of 0 mV). The I–V relationship based on Equation (20) was fitted to the data. This fit determined the magnitude of *P*_*K*_. Figure 6B shows the model I–V when [K^+^]_o_ is an assumed normal [K^+^]_o_ of 5.4 mM, with a corresponding reversal potential of ~−83 mV.

As illustrated in Table 1, the extracellular milieu of the chondrocyte is somewhat unusual, since it has been reported to have a [K^+^]_o_ level of ~7–12 mM. Accordingly, a I–V curve for the 2-pore or TASK K^+^ current was also calculated assuming a [K^+^]_o_ of 15 mM, as shown by the broken trajectory in Figure 6B. We have previously reported (Wilson et al., 2004) that this K^+^ current is strongly inhibited by the anesthetic bupivacaine; and that an effective concentration of bupivacaine resulted in a significant depolarization of the resting potential (see section Discussion and Kindler and Yost, 2005; Webb and Ghosh, 2009).

##### ATP-sensitive K^+^ current: *I*_*K*−*ATP*_

An ATP-sensitive K^+^ current I_K−ATP_ has been identified in chondrocytes that were isolated from the knee joint of a number of different mammalian species (Barrett-Jolley et al., 2010; Mobasheri et al., 2012; Asmar et al., 2016). Our previous experimental work in human articular chondrocytes (Clark et al., 2011) did *not* reveal any significant *I*_*K*−*ATP*_. A likely reason for this is that the intracellular ATP/ADP ratio is set by the “internal pipette solution” in these electrophysiological experiments, and these conditions are such that *I*_*K*−*ATP*_ is unlikely to be activated. However, a relatively low ATP/ADP ratio that is prevalent in the somewhat hypoxic environment of the chondrocyte in articular joints makes it likely that *I*_*K*−*ATP*_ in fact will be activated during physiological biomechanical activity. Thus, a validated but general mathematical expression for this time-independent current (*I*_*K*−*ATP*_) has been included in our mathematical model of the chondrocyte resting potential. This expression scaled to the *I*_*K*−*ATP*_ experimental data published by Mobasheri et al. (2007, 2012). An ATP-dependent K^+^ current simulated using to Equation (21) below is illustrated in Figure S1.

(21) $$\begin{array}{l} I_{K-,ATP}\;=\;\sigma g_{o}p_{o}f_{ATP}\left(V-E_{K}\right) \end{array}$$

where **σ** = 0.6 is the channel density, ***g***_***o***_ is the unitary channel conductance, ***p***_***o***_ = 0.91 is the maximum open channel probability, and ***f*_*ATP*_** is the fraction of activated channels, given by:

(22) $$\begin{array}{l} f_{ATP}=\frac{1.0}{1.0+{\left[\frac{ATP_{i}}{K_{m}}\right]}^{H}} \end{array}$$

(23) $$\begin{array}{l} H=1.3+0.74e^{-H_{K_{ATP}}ADP_{i}} \end{array}$$

(24) $$\begin{array}{l} K_{m}=35.8+17.9{ADP_{i}}^{K_{m,ATP}} \end{array}$$

(25) $$\begin{array}{l} {\text{ADP}}_{\text{i}}\;=\;{\text{C}}_{\text{A}}-{\text{ATP}}_{\text{i}} \end{array}$$

where C_A_ = 8 mM is the total concentration of adenine nucleotides, ADP_i_ and ATP_i_ are the intracellular concentrations of adenosine diphosphate, (ADP) and adenosine triphosphate, (ATP) respectively, H_K,ATP_ = −0.001, and K_m,ATP_ = 0.56.

Note that this ATP-sensitive K^+^ current is *not* utilized in our initial description of the ionic basis for the HAC resting potential. That is, its channel density, **σ**, has been set to zero in our initial or first order model parameterization.

#### Time-independent or background ionic currents

Three distinct time-independent background (or leakage) conductances corresponding to “resting” Na^+^, K^+^, and Cl^−^ fluxes, have been included in this model. Simple mathematical descriptors for each conductance have been formulated, and each yields a linear I–V relationship.

The background (inward) Na^+^ and (outward) K^+^ currents, are described by.

(26) $$\begin{array}{l} I_{Na,b}\;=\;G_{Na,b}\left(V-E_{Na}\right) \end{array}$$

(27) $$\begin{array}{l} I_{K,b}\;=\;G_{K,b}\left(V-E_{K}\right) \end{array}$$

where ***G***_***Na,b***_ = 0.1 nS/pF is the maximum conductance for the background sodium channel, and ***G***_***K,b***_ = 0.07 nS/pF is the maximum conductance for the background potassium channel. [Na^+^]_o_ was initially set to 130 mM under “typical” physiological conditions, but in the environs of the chondrocyte may be in the range shown in Table 1. [Na^+^]_i_, was initially set to 8 mM in this parameterization of the model, and evolves through time, governed by Equation (2), hence E_Na_ also changes with time. Similarly, [K^+^]_o_ was set to “typical” physiological conditions, 5.4 mM, but is likely to be higher in the chondrocyte's environment (Table 1). [K^+^]_i_ was initially set to 140 mM, but this concentration evolved according to Equation (3), thus also changing E_K_.

In mammalian chondrocytes from a number of different species, a significant background Cl^−^ conductance has also been identified (cf. Tsuga et al., 2002; Barrett-Jolley et al., 2010; Funabashi et al., 2010a; Kurita et al., 2015). We have incorporated this type of Cl^−^ current by formulating it as a linear time-independent current, specified by the equations below:

(28) $$\begin{array}{l} I_{Cl,b}\;=\;G_{Cl,b}\left(V-E_{\text{Cl}}\right) \end{array}$$

where ***G***_*Cl,b*_ = 0.05 pS/pF is the maximum conductance for the background Cl^−^ channel, and where the reversal potential, is −65 mV. The I–V relationships for these three background currents are shown in Figure S2.

#### Ion pump and exchanger currents

##### Electrogenic Na^+^/K^+^ pump: *I*_*NaK*_

Active (ATP requiring) extrusion of Na^+^ from chondrocytes is assumed to be achieved by the combined expression level and turnover rate of a conventional electrogenic Na^+^/K^+^ pump. Mobasheri et al. (1997, 1998) have characterized some of the functional properties of an electrogenic Na^+^/K^+^ pump in bovine articular chondrocytes. Our model makes use of the Na^+^/K^+^ pump formulation from Nygren et al. (1998):

(29) $$\begin{array}{l} I_{NaK}={\bar{I}}_{NaK}\left(\frac{{\left[K^{+}\right]}_{o}}{{\left[K^{+}\right]}_{o}+k_{NaK_{K}}}\right)\left(\frac{{\left[Na^{+}\right]}_{i}^{1.5}}{{\left[Na^{+}\right]}_{i}^{1.5}+k_{NaK_{Na}}^{1.5}}\right)\\ \;\left(\frac{V+150}{V+200}\right) \end{array}$$

where ${\bar{I}}_{NaK}$ is the maximal current density 1.58 pA/pF [K^+^]_o_ is the extracellular potassium concentration. It has been initially set to 140 mM for “typical” values; Table 1), [Na^+^]_i_ is the intracellular sodium concentration, as defined previously, and given by equation (2), *k*_*NaK,K*_ is the half-maximum K^+^ binding concentration, and *k*_*NaK,Na*_ is half-maximum Na^+^ binding concentration, with values of 1.0 and 11.0 mmol/L, respectively.

**Figure 7:**
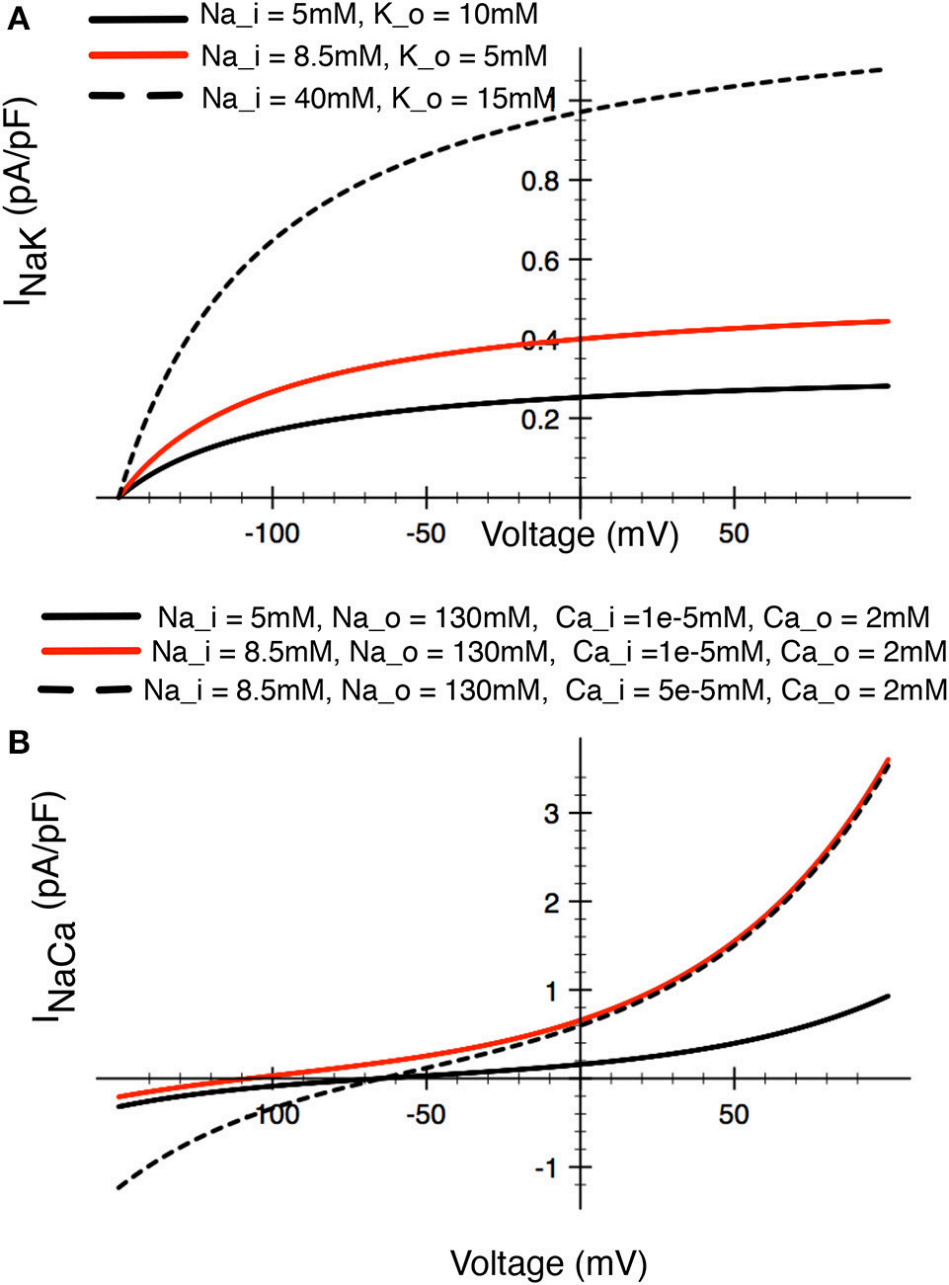
Illustrations of the relative sizes of net currents produced by **(A)** the Na^+^/K^+^ pump, and **(B)** the Na^+^/Ca^2+^ exchanger in a human chondrocyte. The three superimposed I–V curves for the Na^+^/K^+^ pump illustrated in **(A)** bracket the [Na^+^] and [K^+^] levels that have been reported in the literature (see Table 1). The red trace would approximately correspond to baseline conditions for [Na^+^] and [K^+^] in other mammalian cells. The three superimposed I–V curves for the Na^+^/Ca^2+^ exchanger in **(B)** illustrated the relative magnitudes for this current, with the red trace again approximating the resting or baseline I_Na−Ca_ current in most other mammalian cells.

This Na^+^/K^+^ pump activity (the product of expression density and turnover rate) generates a small outward electrogenic current. In this simplified model (cf. Trujillo et al., 1999) this Na^+^/K^+^ pump magnitude has been scaled to achieve a steady-state [Na^+^]_i_ of 10–12 mM. Representative ion transfer relationships for this I_Nak_ are shown in Figure 7A.

##### Na^+^/Ca^2^+ Exchanger: *I*_*NaCa*_

The activity of the Na^+^/Ca^2+^ exchanger plays a key role in Ca^2+^ homeostasis in articular chondrocytes (Sánchez et al., 2006) as it does in most other cell types. We have modeled this electrogenic exchange process using a mathematical expression that we have developed from our work on human atrial myocytes. Its overall properties (ion transfer characteristics, dependence on [Na^+^]_i_ and [Ca^2+^]_i_ have been validated previously (Nygren et al., 1998):

(30) $$\begin{array}{l} I_{NaCa}=k_{NaCa}\frac{{\left[Na^{+}\right]}_{i}^{3}{\left[Ca^{2+}\right]}_{o}e^{\frac{\gamma VF}{RT}}-{\left[Na^{+}\right]}_{o}^{3}{\left[Ca^{2+}\right]}_{i}e^{\frac{(\gamma -1.0)VF}{RT}}}{1.0+d_{NaCa}\{{\left[Na^{+}\right]}_{o}^{3}{\left[Ca^{2+}\right]}_{i}+{\left[Na^{+}\right]}_{i}^{3}{\left[Ca^{2+}\right]}_{o}\}} \end{array}$$

Where *k*_*NaCa*_ is a scaling factor for this current, set to 0.0374842 pA/(mmol/L)^4^, γ is the position of the energy barrier that modulates the voltage dependence of *I*_NaCa_, set to 0.45, and *d*_*NaCa*_ is the denominator constant for the current, set to 0.0003 (mmol/L)^−4.^, [Na^+^]_o_ is the extracellular Na^+^ concentration; [Na^+^]_i_ is the intracellular Na^+^ concentration as given by Equation (2), [Ca^2+^]_o_ is extracellular Ca^2+^ concentration (1.8 mM in a “typical” parameterization; see Table 1), and [Ca^2+^]_i_ is the evolving intracellular calcium concentration given by Equation (4).

This electrogenic ion exchange mechanism has been scaled based on a baseline or resting [Na^+^]_i_ of 12 mM, and [Ca^2+^]_i_ of 3 x 10^−8^ M (see Table 1 and section Discussion). The resulting I–V relationship under these conditions is shown in Figure 7B.

##### Na^+^/H^+^ exchanger: *I*_*NaH*_

Chondrocytes express a Na^+^/H^+^ antiporter (Trujillo et al., 1999; Barrett-Jolley et al., 2010) that contributes importantly to pH regulation. By analogy with its role in many other cells and tissues, this antiporter is responsible for establishing and maintaining the transcellular pH gradient that is essential for maintaining baseline [Na^+^]_i_ levels and optimizing several different intracellular enzyme activities. In addition, intracellular pH indirectly regulates a number of the ion channels that are expressed (e.g., 2-pore K^+^ channels). pH can modulate essential enzymatic processes (e.g., Na^+^/K^+^ pump) in both physiological and pathophysiological settings.

We have used the equations originally developed by Crampin and Smith (2006) to model this electroneutral antiporter:

(31) $$\begin{array}{l} I_{NaH}=N_{NaH}I_{NaH_{mod}}I_{NaH_{exch}} \end{array}$$

(32) $$\begin{array}{l} I_{NaHmod}=\frac{1}{1+(K_{i}^{n_{H}}/{\left[H^{+}\right]}_{i}^{n_{H}}} \end{array}$$

(33) $$\begin{array}{l} I_{NaH_{exch}}=\frac{t_{1}t_{2}-t_{3}t_{4}}{t_{1}+t_{2}+t_{3}+t_{4}} \end{array}$$

(34) $$\begin{array}{l} t_{1}=\frac{k_{1}^{+\;}{\left[Na^{+}\right]}_{o}/K_{Na}^{o}}{\begin{array}{c} 1+\frac{{\left[Na^{+}\right]}_{o}}{K_{Na}^{o}}+\frac{{\left[H^{+}\right]}_{o}}{K_{H}^{o}}\\ \; \end{array}} \end{array}$$

(35) $$\begin{array}{l} t_{2}=\frac{k_{2}^{+\;}{\left[H^{+}\right]}_{i}/K_{H}^{i}}{\begin{array}{c} 1+\frac{{\left[Na^{+}\right]}_{i}}{K_{Na}^{i}}+\frac{{\left[H^{+}\right]}_{i}}{K_{H}^{i}}\\ \; \end{array}} \end{array}$$

(36) $$\begin{array}{l} t_{3}=\frac{k_{1}^{-\;}{\left[Na^{+}\right]}_{i}/K_{Na}^{i}}{\begin{array}{c} 1+\frac{{\left[Na^{+}\right]}_{i}}{K_{Na}^{i}}+\frac{{\left[H^{+}\right]}_{i}}{K_{H}^{i}}\\ \; \end{array}} \end{array}$$

(37) $$\begin{array}{l} t_{4}=\frac{k_{2}^{-\;}{\left[H^{+}\right]}_{o}/K_{H}^{o}}{\begin{array}{c} 1+\frac{{\left[Na^{+}\right]}_{o}}{K_{Na}^{o}}+\frac{{\left[H^{+}\right]}_{o}}{K_{H}^{o}} \end{array}} \end{array}$$

where, *N*_*NaH*_ = 4899, $k_{1}^{+}$ = 10.5, $k_{1}^{-}$ = 0.201, $k_{2}^{+}$ = 15.8, $k_{2}^{-}$ = 183, $k_{H,mod}^{i}$ = 3.07e-5,$K_{H,mod}^{{}^{\circ}}$ = 4.8e-7, $k_{Na}^{i}$ = 16.2, $K_{Na}^{{}^{\circ}}$ = 195, $K_{H}^{i}$ = 6.05e-4, $K_{H}^{{}^{\circ}}$ = 1.62e-3, *n*_*H*_ = 1, *m*_*H*_ = 3, [Na^+^]_i_ and [Na^+^]_o_ are the intracellular and extracellular Na^+^ concentrations as given by Equation (2) respectively. [H^+^]_i_ and [H^+^]_o_ are the intracellular and extracellular proton concentrations. [H^+^]_i_ is an evolving concentration, initialized at pH 7.2. In any given simulation [H^+^]_o_ is selected and then held at this but constant value, typically set to pH 7.4.

#### Intracellular [Ca^2+^]_i_ homeostasis: ATP-dependent Ca pump: *I_*Ca,ATP*_*

In human chondrocytes, [Ca^2+^]_i_ is regulated by a combination of ion transporters, ion pumps, and intrinsic intracellular Ca^2+^ buffering mechanisms. As noted, there is evidence that Na^+^/Ca^2+^ exchanger is functionally expressed in mammalian chondrocytes (Sánchez et al., 2006). Accordingly, Equation (4) in our model that accounts for overall [Ca^2+^]_i_ dynamics includes this antiporter mechanism: an electroneutral, sarcolemmal ATP-requiring Ca^2+^ pump (Nygren et al., 1998), as well as intracellular Ca^2+^ buffering.

The Ca^2+^ pump ion transporter is electroneutral as a consequence of its ability to allow two H^+^ ions to move into the cell for each Ca^2+^ ion that is extruded per transport cycle. We have assumed that the major Ca^2+^ buffer in the cytosol (both in terms of its Ca^2+^ binding capacity and its kinetics) is calmodulin. The cytosolic calmodulin concentration has been adapted from our previous work (Nygren et al., 1998) adjusted for the much smaller intracellular volume of the human chondrocyte. The relevant equations for this electroneutral Ca^2+^ pump and for Ca^2+^ buffering by calmodulin are given by:

(38) $$\begin{array}{l} I_{Ca,ATP}=Imax_{Ca,ATP}\frac{{\left[Ca2+\right]}_{i}}{{\left[Ca2+\right]}_{i}+k_{Ca,ATP}} \end{array}$$

and

(39) $$\begin{array}{l} \frac{dO_{C}}{dt}=2x10^{5}{\left[Ca2+\right]}_{i}(1-O_{C})-476\;O_{C} \end{array}$$

where ***Imax***_*Ca,ATP*_ = 0.6349 pA/pF is the maximal Ca^2+^ pump current density, *k*_*Ca,ATP*_ is the half-maximum Ca^2+^ binding concentration, (0.0002 mmol/L), [Ca^2+^]_i_ is the intracellular calcium concentration as described by Equation (4), and ***O***_*C*_ is the fractional occupancy of the calmodulin buffer by Ca^2+^.

#### Transient receptor potential (TRP) current: *I*_**TRPV*4*_

A fundamental question concerning the electrophysiology of non-excitable cells is: how do they sense the external environment and what ion flux mechanism(s) are responsible for this “trigger/transducer” signal? Ligand-gated cation-selective channels that have properties very similar to those exhibited by some members of the transient receptor potential or TRP family of ion channels (Nilius and Oswianik, 2011; Kaneko and Szallasi, 2014) are expressed in mammalian chondrocytes (cf. Gavenis et al., 2009; Phan et al., 2009; Clark et al., 2010; Asmar et al., 2016). Specifically, TRPV4 is prominently expressed in mouse (Clark et al., 2010) and porcine (Phan et al., 2009) chondrocytes. Previous work on endothelial cells suggests that this type of conductance is the basis for the small Ca^2+^ influx that then “triggers” a much larger release of Ca^2+^ from intracellular stores (the endoplasmic reticulum, Sonkusare et al., 2012) and thus can even produce “Ca^2+^ waves” (Guilak et al., 1999; Han et al., 2012). This important chain of events can initiate dynamic Ca^2+^-dependent intracellular signaling pathways, as well as modulating much longer-term processes such as transcription (Berridge, 1997; Dolmetsch et al., 1997; Berridge et al., 2000; Parekh and Muallem, 2011). Some of these initial sensing/signaling pathways rely on specific integrin isoforms (Wright et al., 1997; Millward-Sadler et al., 2000; Mobasheri et al., 2002; Han et al., 2012).

**Figure 8:**
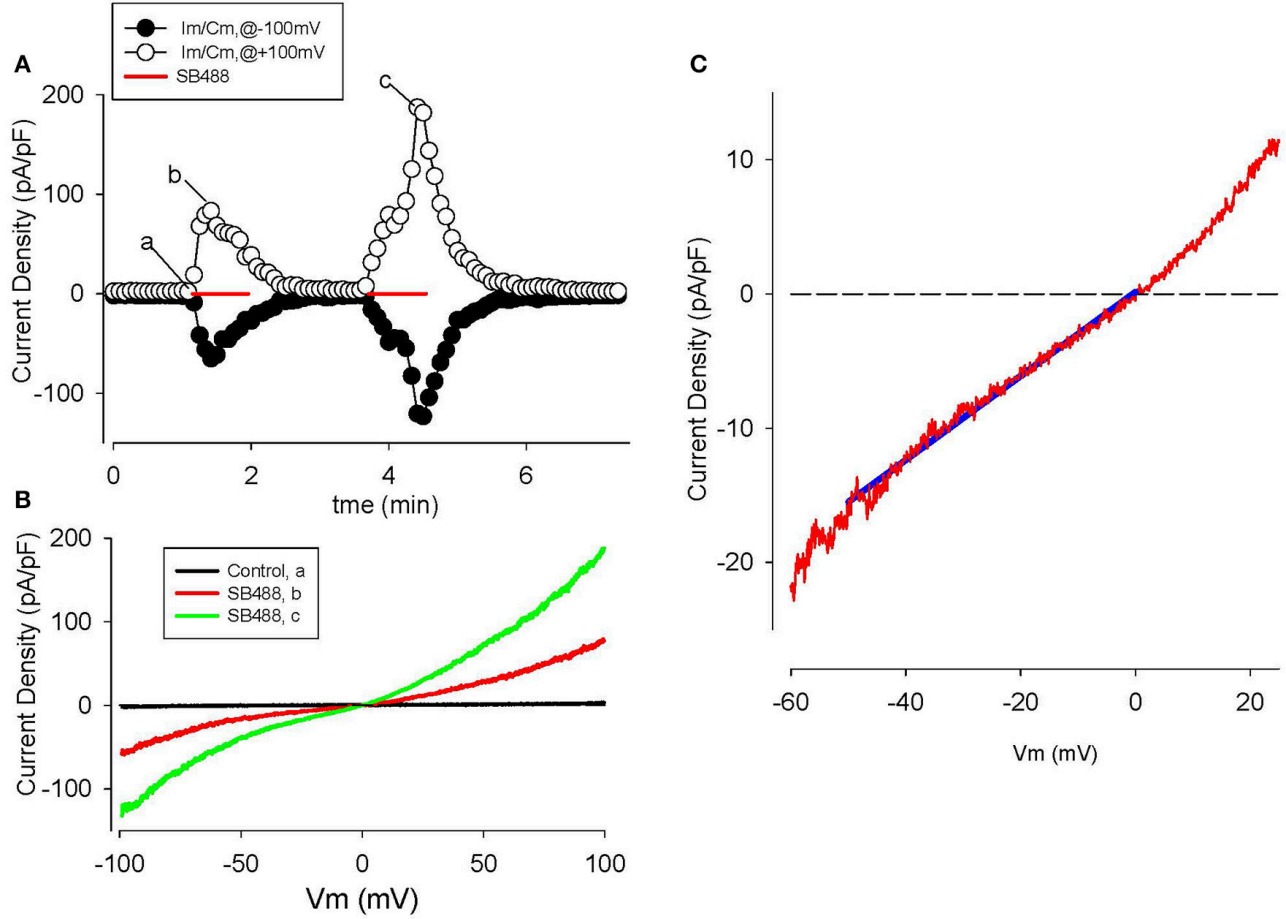
Activation of TRPV4 channel currents by the GSK agonist, SB488, in human articular chondrocytes. **(A)** Time course of membrane current density changes at +100 (open circles) and −100 mV (closed circles) during repeated ramp voltage-clamp protocols. Red bars indicate the application of 1 μM SB488. **(B)** I–V relations before (trace “a,” black) and during application of SB488 (b,c) as illustrated in **(A)**. **(C)** I–V for SB488 induced current on reduced membrane voltage and current scales i.e., in the voltage range of the chondrocyte resting membrane potential. The solid blue line is a linear fit to the I–V over the potential range from 0 to −50 mV.

**Figure 9:**
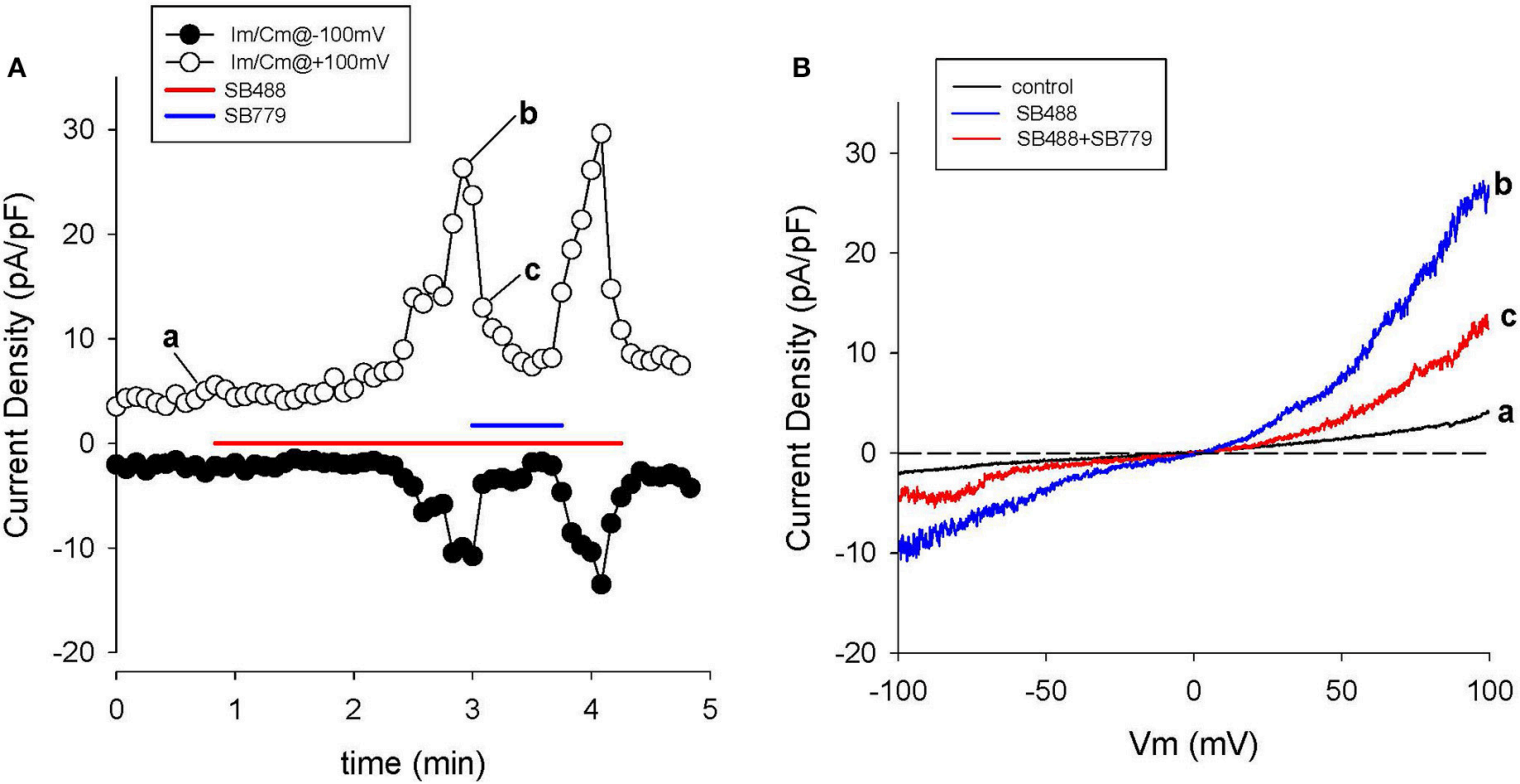
Block of SB488-induced currents through TRPV4 channels by SB779. **(A)** Time course of membrane current changes at +100 (open circles) and −100 mV (closed circles) during repeated ramp voltage-clamp protocols. Colored bars indicate application of 1 μM SB488 (red), or SB488 +1 μM SB779 (blue). Note the nearly complete inhibition of SB488-induced current by SB779. I-V relations before (a), during SB488 (b) and during SB488 + SB779 (c). Panel **(B)** shows 3 superimposed I-V curves: (a) baseline, (b) in the agonist (SB488), and (c) in the combined presence of SB488 and SB779.

We have identified significant TRP currents in human articular chondrocyte (HAC) preparations after superfusion with novel “TRPV4 activator” compounds synthesized by Glaxo Smith Kline (GSK) (Thorneloe et al., 2008; Hilfiker et al., 2013). These experimental results and related mathematical analysis/simulations are shown in Figures 8, 9. Based on this and a variety of other published results we have formulated the working hypothesis that, in the human chondrocyte, there is a multicomponent signaling complex that includes: TRP channels, Ca^2+^-activated K^+^ channels, connexins/pannexins, and purinergic receptors (Loeser et al., 2000; Millward-Sadler et al., 2004; Elliott et al., 2009; Knight et al., 2009; Chekeni et al., 2010; Garcia and Knight, 2010). By analogy with a number of other non-excitable cells this may form the basis for some of the ligand and stretch-sensitive responses in chondrocytes, including the initiation and modulation of intra- and intercellular Ca^2+^ waves.

##### GSK SB488-induced currents in voltage-clamped human chondrocytes

The GSK compound SB488 is a potent agonist for TRPV4 channels (Nilius and Oswianik, 2011; Hilfiker et al., 2013). Our preliminary work has shown that SB488 (1 μM) can induce large non-selective cation currents in primary mouse articular chondrocytes (Giles and Clark, unpublished). Since the inward current due to TRPV4 channels is carried by Ca^2+^ and Na^+^ ions (Nilius and Oswianik, 2011) these non-selective cation channels could constitute one of the essential triggers for intracellular Ca^2+^ release and Ca^2+^-dependent signal transduction in HAC preparations. Figure 8 shows a representative example of the time course and I-V relationships of the SB488-induced currents in a voltage-clamped HAC. Figure 8A consists of a plot of these membrane currents measured at +100 and −100 mV, in response to a ramp voltage-clamp protocol (see Methods section). SB488 (1 μM) application activated a quasi linear current that exhibited both transient and maintained components and declined very slowly after removal of this compound (taking ~1.5 min to return to control levels).

Examples of HAC I-V relations recorded before and during SB488 application are shown in Figure 8B. The baseline or control current was very small and its I–V was essentially linear over the entire potential range that was tested. However, the pipette solution was CsCl-rich, so K^+^ currents were completely blocked. In contrast, after SB488 application a relatively large transmembrane current developed. These two I–V curves intersect very near 0 mV, as shown in the inset. The mean (± s.e.m.) of this reversal potential value for SB488-induced currents in 13 different HAC preparations was 1.8 ± 0.4 mV consistent with the known properties of the TRPV4 family of ion channels.

A different GSK compound, SB779, (Hilfiker et al., 2013) can potently block the currents induced by SB488. It was also studied in our HAC preparations. The data in Figure 9 confirm that SB 799 is an effective blocker of the SB488-induced currents. Figure 9A shows the time course of membrane current at +100 and −100 mV in response to a multiple ramp voltage-clamp protocol. In this HAC, application of SB488 (1 μM) resulted in a slow increase in current i.e., taking about 1 min before currents increased significantly above control levels. This SB488-induced current was quickly blocked by application of SB779 (even in the presence of SB488). This marked reduction of the SB488-induced current by SB779 is consistent with either block of a SB488 “receptor,” or direct, potential-independent block of the SB488-activated ion channels by SB779. Figure 9B illustrates I–V relations recorded at base line, during SB488 alone, and in the presence of SB488 and SB779. The similarity of these I–V relationships and reversal potentials suggest that these compounds do in fact act as selective TRPV4 agonists and antagonists in these HAC preparations.

## Discussion

### The resting membrane potential in human chondrocytes

The new electrophysiological data and first order mathematical model provided by this study add significantly to the previously published papers on fundamental mechanisms of human chondrocyte biology and pathophysiology (cf. Barrett-Jolley et al., 2010; Mobasheri et al., 2012; Asmar et al., 2016). Our main goal was to define the ionic basis for the resting membrane potential, E_m_. Detailed knowledge of the basis for E_m_ of the chondrocyte is especially important since it is a non-excitable cell. In these preparations e.g., endothelial cells, glia, it is known that even very small changes in E_m_ can strongly modulate [Ca^2+^]_i_ (Bouchard et al., 1993; Baczkó et al., 2003; Poon et al., 2014). Either transient or maintained changes in [Ca^2+^]_i_ can modulate Ca^2+^-dependent signaling, as well as the related homeostatic and gene transcription mechanisms (Chao et al., 2006; Lin et al., 2008). In chondrocytes there is also evidence that relatively small alterations in E_m_ can contribute to dynamic regulation of cell volume (Barrett-Jolley et al., 2010; Lewis et al., 2011).

Given the technical difficulty of making accurate, reproducible recordings of transmembrane ionic currents in small cells that have large input resistances, our mathematical model provides an additional basis for understanding the physiological roles of each of the distinct K^+^ currents that have been characterized in human chondrocytes. It is evident from the results in Figures 2–6 that any one, or a combination, of these K^+^ currents could: (i) significantly hyperpolarize the resting potential, and/or (ii) repolarize the chondrocyte after it had been depolarized (Funabashi et al., 2010b). It is also apparent that significant, cyclic depolarization can result from the effects of mechanical activity (stretch or shear), as well as via ligand-gated conductances, (e.g., ATP) or activation of TRP channels.

At this stage of model development our simulations do *not* fully reveal the ionic basis for E_m_ in isolated human chondrocytes. However, they provide further insight into the consistent finding that a range of membrane potential values (−30 to −50 mV) are obtained in single cell patch clamp experiments on human chondrocytes, even after ensuring that ‘multi-GΩ’ seal resistances are obtained. This is because the net outward current that sets the resting membrane potential at steady-state is very small as shown in Figures 2, 7; and is expected from the very large (5–10 GΩ) input resistance (cf. Wilson et al., 2011). Improvement on the work reported here will require extensive additional experimental data, perhaps recorded at physiological temperatures. Additional data analyses and model development will also need to consider alternate approaches for accounting for the interactions among Na^+^/K^+^ pump activity, background Cl^−^ fluxes, and overall cellular osmotic homeostasis (Armstrong, 2003).

### The physiological milieu of the chondrocyte

Classical knowledge of unusual physiologic milieu (see Table 1) within the articular joint yields the expectation that these conditions would be expected to regulate the E_m_, e.g., by altering the conductance or gating properties of intrinsic K^+^ currents that expressed at baseline; through modulation ligand-gated currents; or by changing cell metabolism. For example, the extracellular synovial fluid is somewhat hypertonic (320–340 mOsM vs. approximately 280 mOsM in most mammalian tissues). The effects of osmolarity on voltage gated K^+^ currents have been studied quite extensively, and chondrocytes also express volume-sensitive K^+^ and Cl^−^ currents (Lewis et al., 2011).

**Figure 10:**
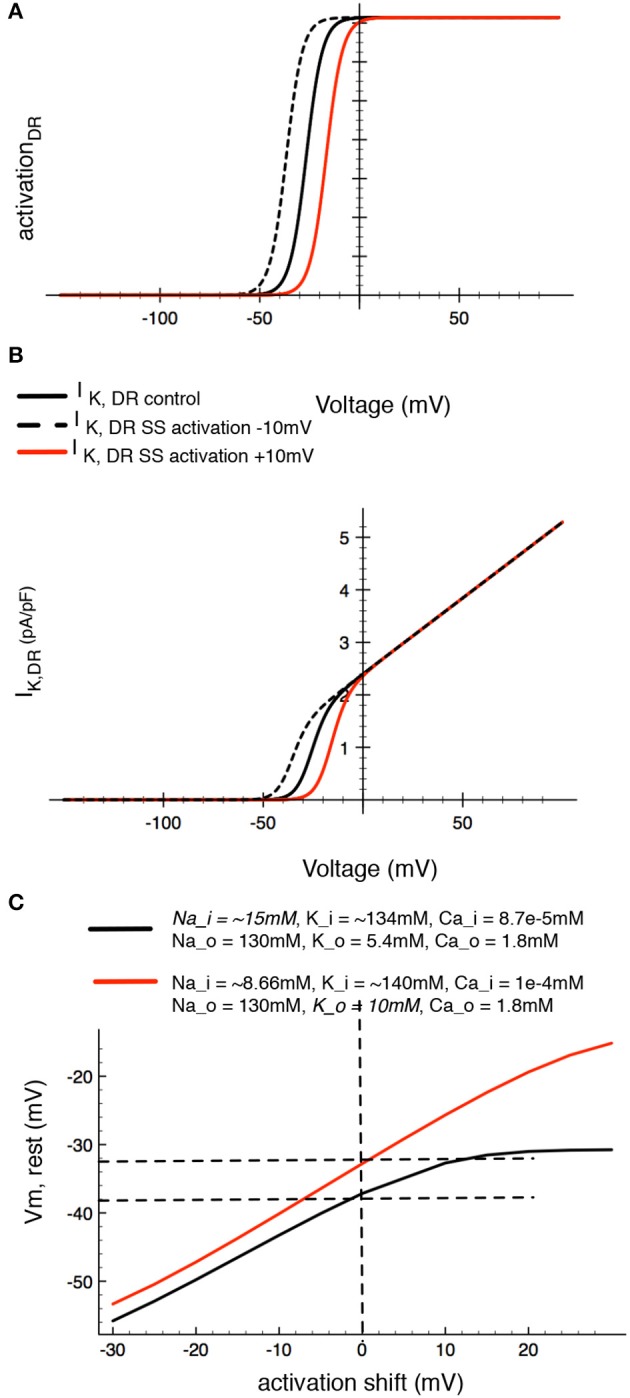
Demonstration of the effects of *in silico* changes in the voltage-dependence for activation of the delayed rectifier K^+^ current, I_K−DR_, Panels **(A,B)**, illustrate the altered activation curves and I-V relationships, respectively. Panel **(A)** shows the derived activation curve for I_K−DR_ (black line) at baseline and after shifting its midpoint ±10 mV to begin an analysis of the effects of changes in surface potential on chondrocyte E_m_. Panel **(B)** shows the (black line computed chondrocyte I–V curves for I_K−DR_ at baseline and after the activation of I_K−DR_ is shifted ±10 mV. Panel **(C)** shows the corresponding changes in resting membrane potential in the baseline model and after [Na^+^]_i_. The red line in **(C)** was computed assuming that intracellular [Na^+^]_i_ is 15 mM. The resulting augmentation/stimulation of the Na^+^/K^+^ pump provides an electrogenic outward current that can significantly hyperpolarize E_m_ (see Discussion section) at all membrane potentials.

We were very interested in an additional mechanism through which changes in the osmolarity of the superfusate or extracellular solution may manifest themselves. It is well-known that changes in osmolarity can alter the “screening or shielding” of discrete membrane surface charges on the plasma membrane and that such changes can regulate channel gating displacement or shifting by altering the voltage-dependence of gating. The main effect of this has been identified as a shift in the voltage-dependence of gating variables, due to altered surface charge or zeta potential (Kell and DeFelice, 1988; Hille, 2001). It is also known that divalent or trivalent cations, (as well as charged osmolytes) can effectively reduce the zeta potential component of the overall membrane potential (Hille, 2001). Although surface potential cannot be measured directly by conventional transmembrane potential recordings, changes in it can be mimicked *in silico* (as shown in Figure 10). Accordingly, in our final set of computations we have shifted the activation curve for *I*_*K*−*DR*_ by 10 mV in either the depolarizing or hyperpolarizing direction (Figure 10A). As expected, this maneuver alters the size of this K^+^ current by changing the fraction that is “available” within the membrane potential range that is near the estimated HAC resting potential (Figure 10B). In the case of the human chondrocyte (as shown in Table 1), surface charge screening resulting from increased osmolarity, would be expected to shift the activation curve to the right (in the depolarized direction), therefore decreasing *I*_*K*−*DR*_ at membrane potentials near E_m_. An important functional consequence would be a tendency to depolarize the resting potential of the chondrocyte (Figure 10C).

Perhaps the strongest evidence that *in situ* the chondrocyte can exhibit significant surface membrane delimited zeta potentials (and restricted diffusion profiles) has been provided by both classical and recent studies that have defined important properties of the chondrocyte pericellular matrix (Poole et al., 1987; Pfander and Gelse, 2007). As mentioned previously, these papers argue in favor of the chondrocyte and its pericellular matrix being defined as an integral functional unit denoted the chondron (Poole et al., 1987; Nguyen et al., 2010; Wilusz et al., 2014). The microanatomy of the chondron, specifically its likelihood for promoting restricted diffusion of e.g., K^+^ and metabolites is likely to be significant factors regulating the chondrocyte “microenvironment.” Within this functional space emerging knowledge of glucose transport (and hence cellular energetics), as well as paracrine and inflammatory factor profiles, will need to be further defined and accounted for (c.f. Mobasheri et al., 1998).

As noted in the Introduction, the pH in the extracellular matrix can be somewhat acidic, ~6.9 as opposed to 7.2 (see Table 1). Changes in [H^+^]_o_ measured in terms of pH alteration can also have “surface change effects” similar to those described above (cf. Hille, 2001). Specifically, lowering pH_o_ (acidification) would result in a decrease in I_KDR_ and therefore an independent and additional tendency to depolarize the chondrocyte resting membrane potential.

Other conditions that characterize the milieu mammalian articular chondrocyte would also be expected to have very significant electrophysiological consequences, mainly by altering E_m_. Perhaps the main one of these in chondrocytes is the significant elevation of intracellular [Na^+^]_i_ levels to (perhaps) as much as 20 mM as opposed to values of 8–10 mM in most other mammalian cells. This elevated [Na^+^]_i_ may partly explain the somewhat atypical pH_i_ values, since intrinsic Na^+^/H^+^ exchange activity and thus pH regulation would be altered. What is likely more important, [Na^+^]_i_ levels in the 15–30 mM range would strongly regulate (activate) the electrogenic Na^+^/K^+^ pump. The resulting increase in outward electrogenic pump current (see Figure 7A) would provide a significant hyperpolarizing influence to the chondrocyte E_m_. This possibility is illustrated by the black traces in Figure 10C. Note that when [Na^+^]_i_ is increased (8.6 to 15 mM) the extra outward Na^+^/K^+^ pump current can significantly hyperpolarize the chondrocyte E_m_. After key principles of chondrocyte Ca^2+^ transport are further understood, it may be possible to revise and improve related aspects of our chondrocyte model as has been done for the PC 12 cell (Duman et al., 2008). One reason for working toward these changes/improvements is the possibility of gaining new insights into [Ca^2+^]_i_ regulated apoptosis and autophagy (Harr and Distelhorst, 2010) as well as senescence (Mobasheri et al., 1998).

### Ca^2+^-influx and Ca^2+^-dependent currents

As noted, activation of TRPV4 channels would be expected to result in a significant influx of Ca^2+^ and Na^+^, and a related depolarization, when the chondrocyte membrane potential is negative to approximately −20 mV, the reversal potential (see Figures 8, 9) for ion flux through these channels. However, any such TRP channel-induced depolarization would be transient, and relatively small. This is because depolarization and/ or Ca^2+^ influx would be expected to be quickly followed by a K^+^ efflux. This would result in repolarization back to E_m_ or even beyond (a hyperpolarization). This K^+^ efflux/outward current would be due to a combination of: (i) the depolarization-induced activation of the delayed rectifier K^+^ currents, *I*_*K*−*DR*_, and/or (ii) turning on of the BK and/or other Ca^2+^-activated K^+^ current (cf. Horrigan and Aldrich, 2002; Magleby, 2003; Sun et al., 2009). In other nonexcitable cells, even a very small Ca^2+^ influx can trigger a large Ca^2+^ release from the ER, thus producing a significant increase in intracellular Ca^2+^, [Ca^2+^]_i_. Moreover, the extent to which [Ca^2+^]_i_ in the ER changes can trigger a secondary but significant net Ca^2+^ influx mediated by I_CRAC_ channels expressed in the surface membrane (c.f. Shaw et al., 2013). In a human chondrocyte-derived cell line, Funabashi et al. (2010b) have shown that histamine can strongly enhance a Ca^2+^-activated K^+^ current, and thus hyperpolarize the chondrocyte membrane potential. This relatively hyperpolarized transmembrane voltage, E_m_, is maintained until [Ca^2+^]_i_ and/or Ca^2+^-dependent signaling mechanisms reset to “resting” values. This Ca^2+^ dependent hyperpolarization may also contribute to chondrocyte differentiation (Muramatsu et al., 2007).

### Connexin-mediated current flow and electrotonic interactions

As mentioned in the Introduction, chondrocytes in articular joints from adult humans function as isolated, single cells. Interestingly, however, Cell Physiological data from early adolescent articular joint preparations suggest that the growth plate of articular joints includes small groups of closely opposed chondrocytes. In these preparations, expression of selected members of the connexin family of intercellular communication proteins (e.g., Cx43) has been detected using standard immunohistochemical approaches (Chi et al., 2004; Mayan et al., 2013a,b; Asmar et al., 2016).

We also note that is Cx43 increased expression in isolated adult chondrocytes can result in release ATP in response to e.g. mechanical perturbations (Millward-Sadler et al., 2004; Knight et al., 2009; Garcia and Knight, 2010). One plausible mechanism for this, is the occurrence of transient openings of these “hemi-channels” that consist of either pannexin or connexin subunits (Saez et al., 2003; Garcia and Knight, 2010; Penuela et al., 2013). Given this important functionality, our mathematical model also includes a connexin-mediated conductance (Figure 4). However, since this initial model development was focused on simulating adult chondrocyte behavior under a restricted set of physiological conditions, these connexin single-cell channels were shut off (assigned a conductance value of 0 pS) in all simulations that form the basis of this paper. We acknowledge that this choice does **not** allow this model to account for any of the interesting function properties that arise from the so-called connexon-43 hemichannel behavior in chondrocytes (Knight et al., 2009). This “hemichannel activity” in chondrocytes can also be mediated by the pannexin family of integral membrane proteins (Bond et al., 2011; Matta et al., 2015).

### Limitations of the mathematical model of the human chondrocyte resting membrane potential

The mathematical model that we have developed is an important advance. However, we recognize that it has significant limitations. These include:

- It is apparent that Ca^2+^ is an essential signaling molecule in the chondrocyte. Expression of L-type Ca^2+^ channels has been reported in growth plate chondrocytes (Sugimoto et al., 1996; Zuscik et al., 1997), but not in other single cell adult chondrocyte preparations. In the future, more detailed consideration of the details of (i) Ca^2+^ channels (ii) TRP channels (iii) CRAC channels (iv) the Na^+^/Ca^2+^ exchanger and (v) the Ca^2+^ pumps in both the endoplasmic reticulum and the surface membrane must be included in the model. In fact systematic experimental and modeling studies of each functional element in chondrocyte [Ca^2+^] homeostasis are needed, perhaps with an emphasis on the so-called Ca^2+^ signalosome with emphasis on the Ca^2+^ pump in the endoplasmic reticulum, (Kranias and Hajjar, 2012).
- Mathematical expressions that would allow simulations of what has been termed “the AM and FM modes of Ca^2+^ signaling” (Berridge, 1997; Berridge et al., 2000), will require consideration of [Ca^2+^]_i_-dependent phosphorylation and dephosphorylation reactions, the involvement of IP-3 in Ca^2+^ release, (Mak and Foskett, 2015) as well as accounting for the Ca^2+^-dependence involved in transcriptional regulation of ion channel, antiporter and pump target molecules.
- There is evidence that cell culture conditions can alter both chondrocyte phenotype and gene expression profiles (Spitzer et al., 2000; Chen et al., 2012; but see Asmar et al., 2016). These patterns of changes will need to be taken into account for attempting to either interpolate or extrapolate findings from this model. This limitation can be addressed when new data sets are available from isolated chondrocytes that have been isolated from defined ‘zone’ (Berridge, 2007; Amanatullah et al., 2014) cultures in 3-D scaffold or substrates with known stiffness (Chen et al., 2012).

## Summary

This mathematical model of chondrocyte electrophysiology provides a reliable platform for integrating and evaluating both recent and well-established experimental data that is relevant to the generation of the resting potential. At a minimum, given that the chondrocyte is in a unique but relatively inaccessible, environment, our model provides new insights into: the biophysical effects of alterations in ionic strength of synovial fluid on ion channel voltage-dependent gating (zeta potential effects). Key elements of the zeta potential working hypothesis can be tested if recent advances in voltage-dependent dye methods are implemented to provide a means of separating the various components of the signal that underlies the “transmembrane potential” in small nonexcitable cells (Cohen and Venkatachalam, 2014). It will also be necessary to account for the effects of cyclic stretch on chondrocyte ion channels by defining the main strain dependent alterations in channel gating voltage dependence or kinetics (cf. Hille, 2001; Maleckar et al., 2009). Approaches for detecting and determining the limitations of present patch clamp methods that can bias key electrophysiological data sets and influence their interpretation must continue to be utilized and refined. However, even in its present form this first-order model will continue to be useful for rationalizing and bringing together genomic data from microarray expression profiles, and understanding ion channel/antiporter drug target initiatives.

## Author contributions

RBC carried out all of the experimental work in this paper, and provided valuable input into the development and validation of the mathematical model. MM when supervising Dr. H. Narayanan at Simula Research Inc in Oslo, Norway lead the mathematical modeling component of this study. BV, a Senior Scientist at Glaxo Smith Kline, Philadelphia, USA provided valuable input into experimental design and facilitated transfer of the GSK properiarty cell lines and pro-drugs. WRG lead the project and wrote the manuscript. All authors have read and revised the manuscript, and are in agreement with its main findings and conclusions.

### Conflict of interest statement

The authors declare that the research was conducted in the absence of any commercial or financial relationships that could be construed as a potential conflict of interest.
