# Supplementary material for: The Resting Potential and K+ Currents in Primary Human Articular Chondrocytes
Source: Front Physiol. 2018 Sep 4;9:974. doi: 10.3389/fphys.2018.00974 (PMC6131720; doi:10.3389/fphys.2018.00974)
Supplement: Supplementary file 1 [file Data_Sheet_1.docx]

###

### Figure S-1. Linear time-independent or background I-V relationships for Na^+^, K^+^, and Cl^-^ in a human chondrocyte. Individual ion selective conductances are shown as: Na^+^ (red), K^+^ (blue) and Cl^-^ (green). The combined net background current is scaled to yield the experimentally determined value of chondrocyte input resistance at approximately -40 mV.

**
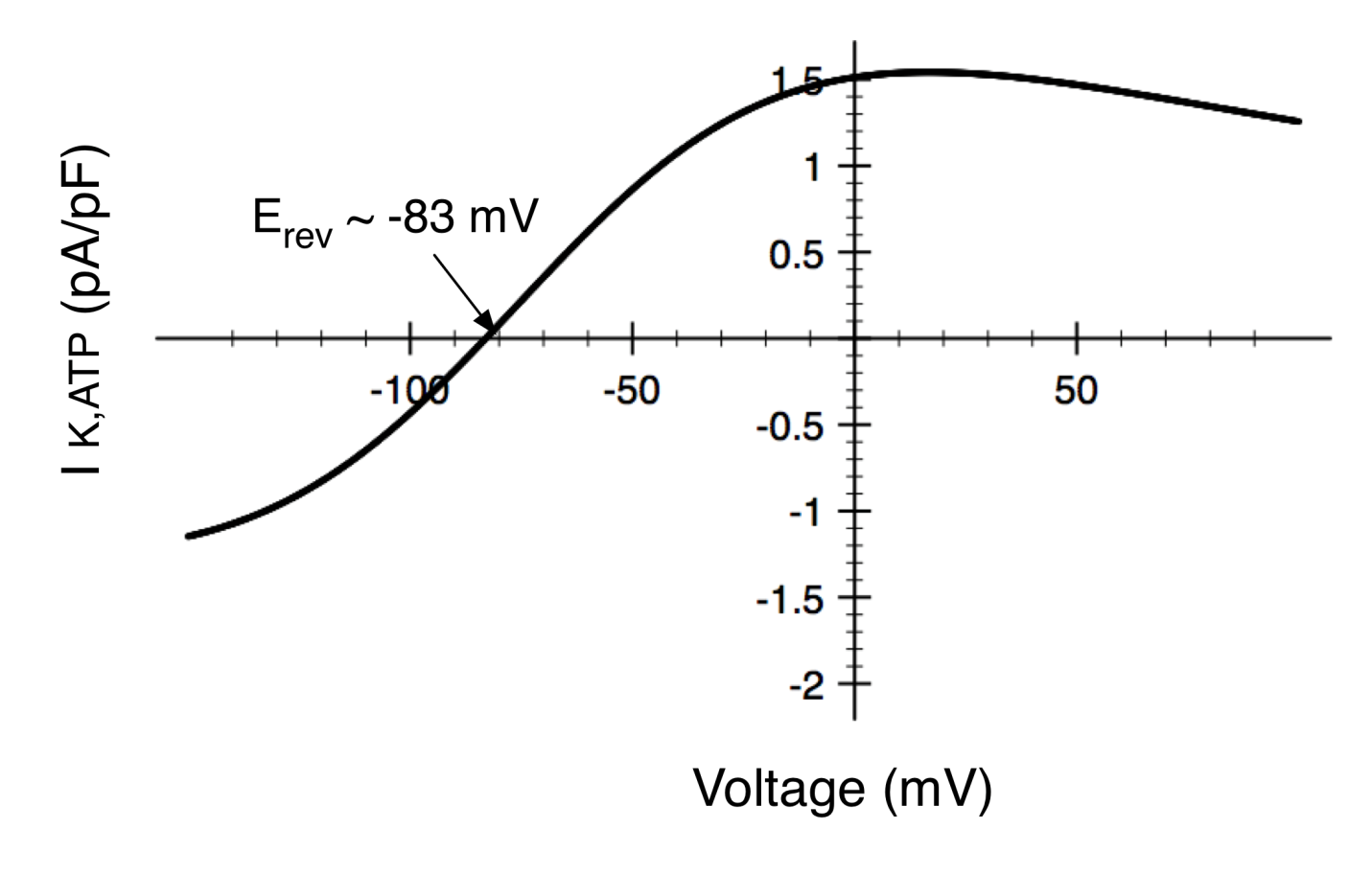
**

**Figure S-2.**ATP-sensitive K^+^ current, I_K-ATP_, in an isolated chondrocyte. Panel A shows a typical I-V relationship for this K^+^ current, scaled to provide an outward current density that would be consistent with those for I_K-DR_ and I_K-Ca_ in human chondrocytes. This I-V curve was computed using a standard mathematical formulation for this current that is described in the text.
